# Unexpected structural isomers of AlFe_2_O_4_^+^ and AlCo_2_O_4_^+^: vibrational spectroscopy and ion mobility combined with quantum chemistry†

**DOI:** 10.1039/d5sc02681d

**Published:** 2025-05-19

**Authors:** Winni Schwedland, Tatiana C. Penna, Henning Windeck, Fabian Müller, Stephen Leach, Joachim Sauer, Xavier R. Advincula, Fabian Berger, Nanako Ishida, Keijiro Ohshimo, Fuminori Misaizu, Ya-Ke Li, Arghya Chakraborty, Francine Horn, Knut R. Asmis

**Affiliations:** a Institut für Chemie, Humboldt-Universität zu Berlin Unter den Linden 6 10099 Berlin Germany js@chemie.hu-berlin.de; b Yusuf Hamied Department of Chemistry, University of Cambridge Lensfield Rd Cambridge CB2 1EW UK fb593@cam.ac.uk; c Wilhelm-Ostwald-Institut für Physikalische und Theoretische Chemie, Universität Leipzig Linnéstraße 2 04103 Leipzig Germany knut.asmis@uni-leipzig.de; d Fritz-Haber-Institut der Max-Planck-Gesellschaft Faradayweg, 4-6 14195 Berlin Germany; e Graduate School of Science, Tohoku University 6-3 Aoba, Aramaki, Aoba-ku Sendai 980-8578 Japan misaizu@tohoku.ac.jp

## Abstract

The structure and reactivity of the mixed metal oxide clusters Al_2_MO_4_^+^ and AlM_2_O_4_^+^ (M = Fe, Co), formally obtained by transition metal ion substitution from Al_3_O_4_^+^, are studied using infrared photodissociation (IRPD) spectroscopy, ion-mobility mass-spectrometry (IM-MS) and quantum chemistry. We use density functional theory (DFT) in combination with global structure optimization to identify low energy structures and to connect them to the IRPD and IM-MS data. Insights into anharmonic and temperature effects are obtained from machine learning-based molecular dynamics simulations. While all metal ions are equal in the cone-shaped structure of M_3_O_4_^+^, the mixed metal oxide clusters attain different, more stable structures, in which the metal ions are either in different oxidation states (Al_2_MO_4_^+^) or have different coordination numbers (AlM_2_O_4_^+^). The present results illustrate that different DFT functionals may accurately describe local minimum structures, but reliable relative energies of isomers with differently coordinated transition metal ions require multi-reference wavefunction calculations.

## Abstract 2

Isovalent metal substitution in metal oxides induces structural changes that are much larger for gas phase clusters than for the corresponding bulk oxides.

## Graphical abstract

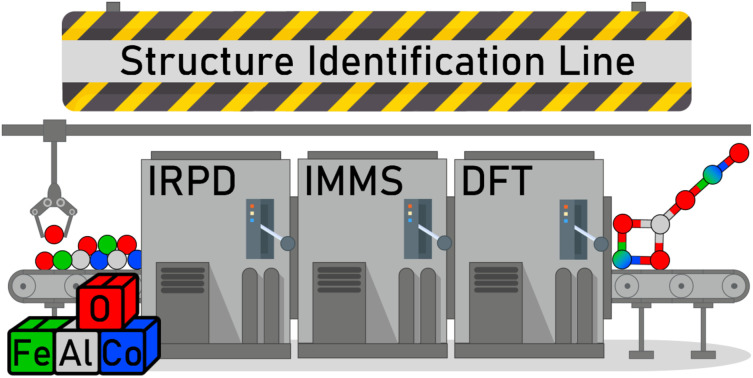

## 1 Introduction

Corundum (α-Al_2_O_3_) and hematite (α-Fe_2_O_3_) belong to Earth's most ubiquitous metal oxides. Together, they play important roles in geochemical processes, as industrial materials, and in catalysis.^1–3^ For example, Al^3+^-substituted hematite nanoparticles represent an important active component in soils, affecting the sequestration and bioavailability of contaminants. However, how the Al-substitution-induced morphological changes affect the adsorption behavior of hematite for contaminants remains poorly understood.^4^

Both materials, corundum and hematite, share the same rhombohedral crystal structure, in which a metal ion M^3+^ binds to six O^2−^ ions to form an octahedron. The isomorphous substitution of Fe^3+^ by Al^3+^ in hematite is well documented and there is much interest in understanding how the presence of the smaller, less electronegative Al^3+^ ions affect the material properties.^1,5^ Strain is induced upon Fe^3+^-substitution as a consequence of the smaller lattice parameters for α-Fe_2_O_3_ compared to α-Al_2_O_3_. Hence, substitution is limited to about 8%, leading to the well-known miscibility gap of the corundum-hematite system.^6^ Intermediate orthorhombic AlFeO_3_ can be prepared at temperatures above 1300 °C but is unstable at 298 °C (with respect to the pure phases).^7^ Recently, morphology variations of hematite crystals by Al-substitution have been studied by electron microscopy and motivated subsequent investigations using density functional theory (DFT).^1,8^ Still, the impact of Al-substitution on the structural and electronic properties of hematite remains ill-characterized, and tools that allow predicting such properties reliably are in demand.

Metal oxide clusters isolated in the gas phase represent well-defined model systems that can be studied using the highly sensitive and selective tool kit of action spectroscopy.^9–11^ Cryogenic ion vibrational spectroscopy in combination with electronic structure calculations yields important structural information and allows to analyze structure–reactivity correlations.^12–14^ Additional information about the presence of different structural isomers can be obtained from ion-mobility measurements.^15,16^

**Scheme 1:**
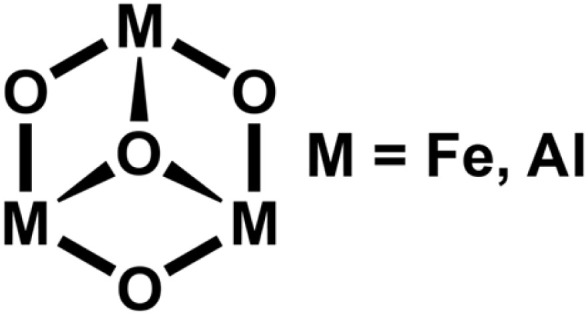
Structure of M_3_O_4_^+^.

The simplest positively charged cluster models for pure alumina and pure hematite are Al_3_O_4_^+^ and Fe_3_O_4_^+^, respectively. They are isoelectronic with the solid materials, with all metal and O ions in their bulk oxidation states of +3 and −2, respectively. Both Al_3_O_4_^+^,^17^ and Fe_3_O_4_^+^,^18^ have “cone”-like structures with a central trivalent oxygen ion (Scheme 1).

**Scheme 2:**
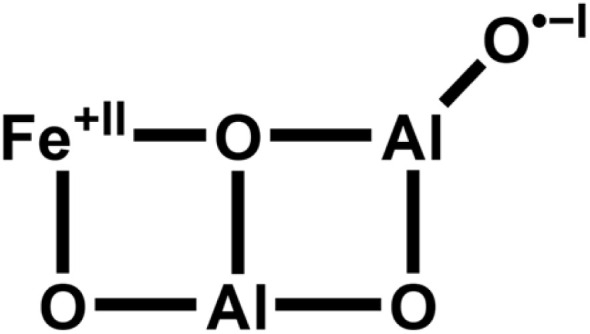
Structure of FeAl_2_O_4_^+^.

Here, we examine the substitution of a transition metal (M = Fe, Co) by an Al ion in M_3_O_4_^+^, yielding AlM_2_O_4_^+^, and the substitution of Al by a transition metal ion in Al_3_O_4_^+^, yielding Al_2_MO_4_^+^. We are particularly interested in the question whether the substitution is isomorphous and, if not, which factors drive the structural changes and how do such changes affect the reactivity towards methane. It is known that substitution of Al by Fe in Al_3_O_4_^+^ is not isomorphic and that a change of the oxidation states from Fe^+III^/O^−II^ to Fe^+II^/O˙^−I^ is accompanied by the change to a planar bicyclic structure (Scheme 2).^19^ The terminal oxygen radical site explains why Al_2_FeO_4_^+^ abstracts hydrogen from methane,^19^ whereas Al_3_O_4_^+^ is unreactive towards methane.

Specifically, we use infrared photodissociation (IRPD) spectroscopy to measure vibrational spectra and employ ion-mobility mass-spectrometry (IM-MS) measurements to distinguish between differently shaped isomers. Additionally, we use DFT in combination with global structure optimization to identify low energy structures and to connect them to the experimental IRPD spectra and IM-MS data. The reactivity of the clusters towards methane is measured in an ion trap and compared to a DFT-based reaction energy profile.

## 2 Experimental results

### 2.1. Mass spectrometry

The metal oxide cations are produced by laser vaporization of a single mixed-metal rod (see the Methods section for details). The ablation of metal ions of two different elements yields more congested mass spectra compared to those metal oxide cations which contain only one metal type^20^ and, consequently, more stringent settings are required to ensure that only the cation of interest is probed. First, unit mass resolution was used for measuring the IRPD spectra. Second, the possibility of forming multiple isobaric species needs to be considered. While no obvious overlap is apparent for Al^56^Fe_2_^16^O_4_^+^ (*m*/*z* 203), the only naturally occurring Co isotope (^59^Co) is isobaric with ^27^Al^16^O_2_. Therefore, the latter experiments were performed on Al_2_Co^18^O_4_^+^ (*m*/*z* 185) and AlCo_2_^18^O_4_^+^ (*m*/*z* 217) using molecular ^18^O to avoid ion signal overlap with Al_3_^16^O_6_^+^(*m*/*z* 177), Al_2_Co^16^O_6_^+^ (*m*/*z* 209) and Al_3_^16^O_8_^+^ (*m*/*z* 209). The mass spectra obtained in this way are shown in Fig. S1.†

### 2.2. Ion-trap reactivity

We performed ion-trap reactivity measurements towards methane under multiple collision conditions to test for the presence of reactive sites (see Fig. S2†). These measurements show that the doubly substituted cations AlFe_2_O_4_^+^ and AlCo_2_O_4_^+^ are not reactive towards methane, similar to Al_3_O_4_^+^.^21^ In contrast, the singly substituted cations Al_2_FeO_4_^+^ and Al_2_CoO_4_^+^ readily react with methane, with H atom abstraction efficiencies of 54% and 75%, respectively. Previously, the corresponding analogs Al_2_MO_4_^+^ (M = Zn, Ni) have been found to abstract an H atom from methane under formation of a methyl radical.^21,22^

### 2.3. Vibrational action spectroscopy

To obtain structural information on the mixed metal oxide cations we measured vibrational action spectra through IRPD spectroscopy of the corresponding He-tagged species.^23^ This method provides vibrational spectra in the linear absorption regime, typically a prerequisite for the unambiguous structure assignment based on a comparison to calculated infrared (IR) frequencies and intensities.^14^

**Fig. 1:**
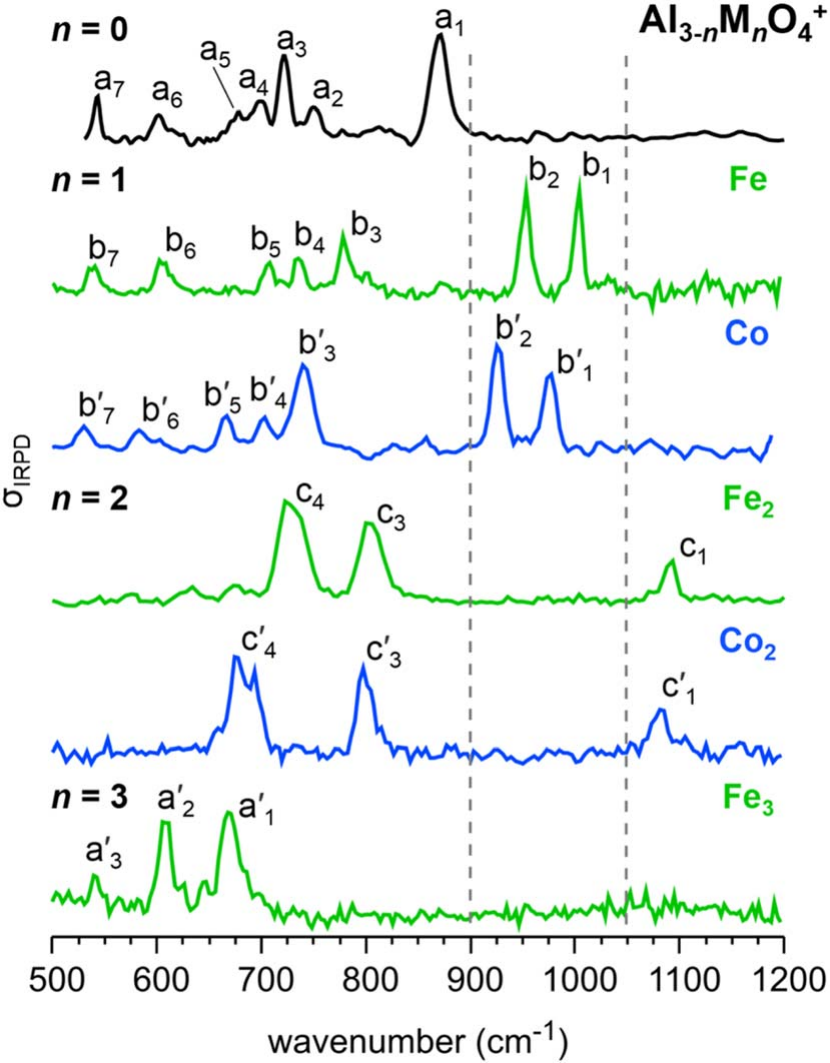
IRPD spectra (from top to bottom) of Al_3_O_4_^+^(He_3_),^17^ Al_2_FeO_4_^+^(He_1,2_),^19^ Al_2_Co^18^O_4_^+^(He_1,2_), AlFe_2_O_4_^+^(He_1,2_), AlCo_2_^18^O_4_^+^(He_1,2_), and Fe_3_O_4_^+^(He).^24^ See Table 1 for band positions and assignments. Characteristic spectral regions are separated by the broken lines.

Fig. 1 compares the IRPD spectra of the metal oxide cations Al_3_O_4_^+^ (top panel) and Fe_3_O_4_^+^ (bottom panel), which exhibit similar structures of *C*_3v_ symmetry,^17,18,24–26^ as well as the spectra of the mixed metal oxide cations Al_3−*n*_M_*n*_O_4_^+^ (M = Fe, Co; *n* = 1, 2) in the spectral range from 500 to 1200 cm^−1^. Band positions and assignments are summarized in Table 1. While Fe_3_O_4_^+^ and Al_3_O_4_^+^ share the same structure type, their IRPD spectra are shifted and have a different appearance. This is attributed to a combined effect of the higher atomic mass of the metal ions and the weaker bonds in Fe_3_O_4_^+^ (see Fig. S4†).

**Table 1:** Experimental IRPD-derived vibrational band positions of He-tagged Al_3−*n*_M_*n*_O_4_^+^ and TPSSh/def2-TZVPP harmonic vibrational wavenumbers of the corresponding bare cations in cm^−1^ with relative IR intensities in parenthesis and the assignment of vibrational modes. The vibrations are categorized in symmetric (s), antisymmetric (as) or antiphase (ap) as well as in stretching (*ν*), and bending (*δ*) modes with O^t^ denoting a terminal oxygen ion^a^

*n*		Experiment	Calculated		Experiment	Calculated	Assignment
**0**	**Al**						
	a_1_	871 (100)	846 (100)				*ν*_as_(O–Al–O)
	a_2_	750 (30)	730 (27)				*ν*_s_(O–Al–O)
	a_3_	722 (79)	697 (41)				*ν*_s_(O–Al–O)
	a_4_	700 (36)	692 (60)				*ν*_s_(O(–Al)_3_)
	a_5_	678 (25)					
	a_6_	602 (23)	576 (12)				*ν*_s_(O(–Al)_3_)
	a_7_	543 (40)	532 (7)				*ν*_as_(O(–Al)_3_)

**1**	**Fe**			**Co**			
	b_1_	1004 (100)	986 (80)	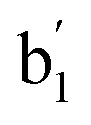	976 (72)	953 (62)	*ν*_as_(O–Al–O)
	b_2_	953 (100)	936 (100)	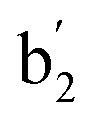	926 (100)	913 (100)	*ν*_s_(Al–O^t^)
	b_3_	778 (56)	763 (76)	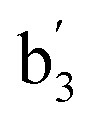	740 (82)	725 (65)	*ν*_ap_(M–O–Al)
	b_4_	735 (34)	724 (18)	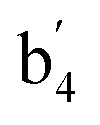	703 (31)	687 (19)	*ν*_s_(O–Al–O)
	b_5_	708 (30)	690 (19)	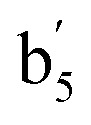	664 (33)	650 (12)	*ν*_s_(O–Al–O)
	b_6_	602 (32)	599 (25)	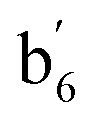	583 (19)	569 (21)	*ν*_as_(O–M–O)
	b_7_	541 (27)	539 (32)	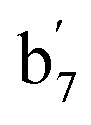	531 (23)	528 (27)	*δ*(M–O–Al)

**2**	**Fe**_**2**_			**Co**_**2**_			
	c_1_	1091 (28)	1097 (100)	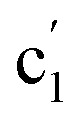	1082 (47)	1067 (100)	*ν*_ap_(Al–O–M)
	c_2_		893 (3)	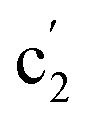		839 (1)	*ν*(M–O^t^)
	c_3_	803 (75)	760 (9)	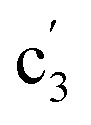	798 (57)	714 (8)	*ν*_s_(O–M–O)
	c_4_	727 (100)	689 (11)	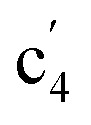	681 (100)	650 (9)	*ν*_s_(O–Al–O)
			687 (11)			646 (6)	*ν*_as_(O–M–O)

**3**	**Fe**_**3**_						
	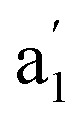	669 (100)	678 (95)				*ν*_as_(O–M–O)
	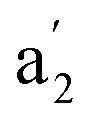	609 (91)	641 (100)				*ν*_s_(O–M–O)
	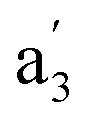	542 (39)	527 (55)				*ν*_s_(O(–M)_3_)

a The values for Al_3_O_4_^+^, Al_2_FeO_4_^+^ and Fe_3_O_4_^+^ are from ref. 17, 19 and 24, respectively. Experimental relative intensities are determined from the ratios of the integrated peak areas.

There are two main observations: (i) the spectra of the mixed metal oxide cations for the same *n* but different transition metals are very similar, suggesting identical structural motifs with the remaining differences being due to slightly different masses and bond strengths. (ii) The IRPD spectra for different *n* are so different that substantial structural changes are very likely upon Al/M (or M/Al) substitution. In other words, there are no indications for isomorphous substitution, neither for substitution of Al by M (*n* = 0 → 1), nor for substitution of M by Al (*n* = 3 → 2).

The IRPD spectra for *n* = 1 shown in Fig. 1 have seven bands (Fe: b_1_ to b_7_; Co: b′_1_ to b′_7_), with similar relative positions and relative intensities for Fe and Co (see Table 1). The IRPD bands in the Al_2_Co^18^O_4_^+^ spectrum are slightly red-shifted (up to 25 cm^−1^) with respect to the corresponding IRPD bands of Al_2_FeO_4_^+^, which can be explained by the higher mass of the O isotope in the Co-containing ions. As reported previously, the replacement of one Al by either an Fe ion^19^ or a Ni ion^21^ leads to a change from the “cone”-like structure with a central trivalent O ion (*C*_3v_ symmetry) to a planar bicyclic frame (*C*_s_ symmetry) with a terminal Al–O˙^−I^ radical site. The Al_2_MO_4_^+^ spectra suggest that a similar structural change occurs for the substitution with a Co ion.

In analogy to Al_2_MO_4_^+^, the IRPD spectra of the two AlM_2_O_4_^+^ cations are very similar to each other, exhibiting three characteristic absorption features (Fe: c_1_ to c_4_; Co: c′_1_ to c′_4_). The bands in the Co-substituted spectrum are again slightly red-shifted due to the presence of ^18^O. The AlM_2_O_4_^+^ IRPD spectra differ from those of all other compositions by a characteristic band (Fe: c_1_; Co: c′_1_) above 1050 cm^−1^. Moreover, they exhibit no bands between 900 and 1050 cm^−1^ or between 500 and 650 cm^−1^, which are the spectral regions where prominent features are observed for Al_3_O_4_^+^, Al_2_MO_4_^+^, and M_3_O_4_^+^.

### 2.4. Ion mobility measurements

**Fig. 2:**
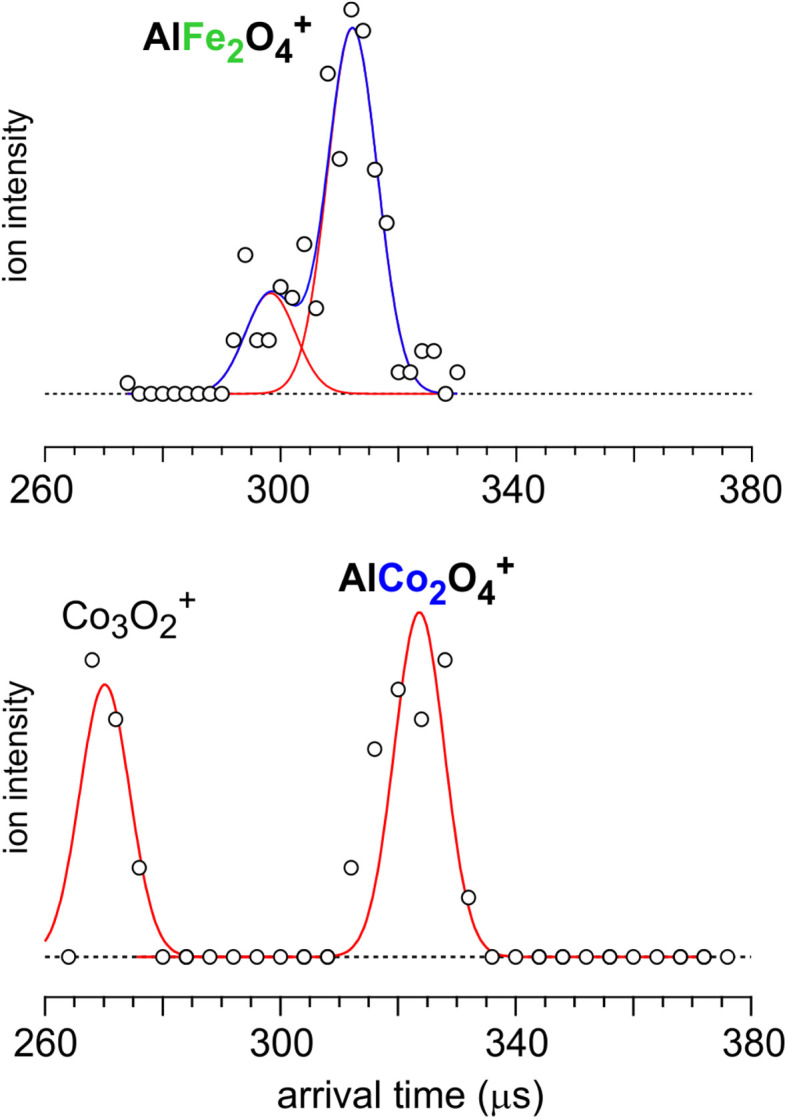
Arrival time distributions of AlFe_2_O_4_^+^ and AlCo_2_O_4_^+^ in the ion mobility measurement. Red solid curves are Gaussian line shape functions that are used for fitting the experimental plots (black circles). The width of the Gaussian line shape function was determined from the experimental resolution of our IM-MS apparatus. Blue solid curves are the sum of two Gaussian line shape functions. The ion signal of Co_3_O_2_^+^ is also observed due to the same mass (*m*/*z* 209).

Additional structural information is obtained from IM-MS measurements,^15,16^ which allow discerning structural isomers based on shape differences. These measurements reveal a bimodal feature in the arrival time distribution of AlFe_2_O_4_^+^, with an intensity ratio of 0.3 : 1, and a single peak in the arrival time distribution of AlCo_2_O_4_^+^ (see Fig. 2). From the peak position of the bimodal distribution in Fig. 2, the AlFe_2_O_4_^+^ collision cross sections (CCSs) were determined to be 67.0 ± 0.9 and 70.6 ± 0.9 Å^2^, and 71.4 ± 0.8 Å^2^ for AlCo_2_O_4_^+^ (error bars were determined from eight independent measurements). We also studied monosubstituted Al_2_FeO_4_^+^ for which a single peak is observed in the arrival time distribution corresponding to a CCS of 68.4 ± 1.1 Å^2^ (see Fig. S3†).

## 3 Computational results

### 3.1. Isomer structures and harmonic IR spectra

**Fig. 3:**
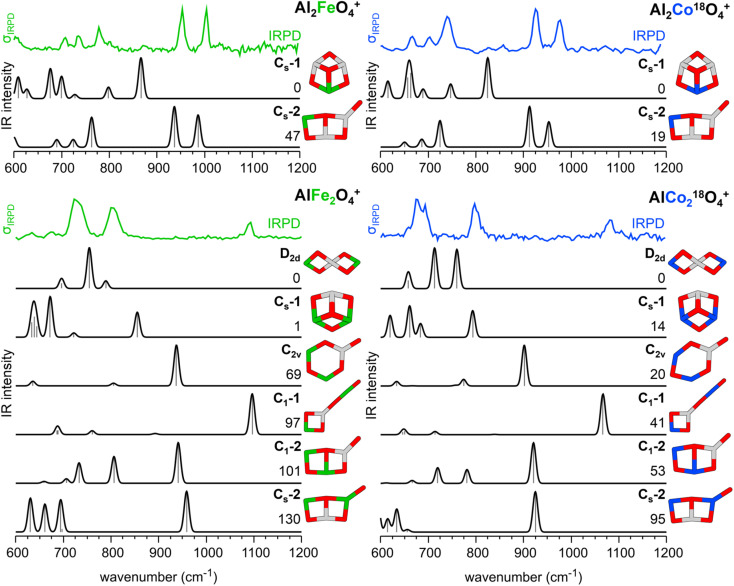
IRPD spectra of Al_2_FeO_4_^+^(He_1,2_),^19^ Al_2_Co^18^O_4_^+^(He_1,2_), AlFe_2_O_4_^+^(He_1,2_), and AlCo_2_^18^O_4_^+^(He_1,2_) as well as the corresponding harmonic IR spectra of the bare cations as calculated with TPSSh/def2-TZVPP for their high spin states. For each composition, different isomers are considered, and the respective point groups are reported. Relative energies (including zero-point vibrational energies) are given in kJ mol^−1^. Color code: O – red, Al – gray, Fe – green, and Co – blue.

The energetically most stable structures of Al_2_CoO_4_^+^, AlFe_2_O_4_^+^, and AlCo_2_O_4_^+^ were identified using a genetic algorithm^27,28^ with DFT for the respective high spin states. The most stable isomers were locally reoptimized with the TPSSh^29^ exchange-correlation functional and the def2-TZVPP^30,31^ basis set. The same method was used to calculate IR spectra (wavenumbers and intensities) using the (double) harmonic approximation. Analogous to Al_2_FeO_4_^+^, the oxidation states of the Al_2_CoO_4_^+^ isomers C_s_-1 and C_s_-2 are Co^+III^/O^−II^ and Co^+II^/O˙^−I^, respectively. The oxidation states for all isomers of the composition AlM_2_O_4_^+^ shown in Fig. 3 are M^+III^/O^−II^, except for C_2v_ and C_1_-2 which also contain an O radical with M^+II^/O˙^−I^.

Fig. 3 compares the IRPD spectra of the He-tagged clusters Al_2_FeO_4_^+^,^19^ Al_2_CoO_4_^+^, AlFe_2_O_4_^+^, and AlCo_2_O_4_^+^ to calculated harmonic IR spectra of different isomers of the bare cations. He-tagging, which is experimentally necessary to measure IRPD spectra in the linear absorption regime, does not significantly affect the calculated IR spectra (see Fig. S9†) and is therefore neglected in the further discussion.^19^

#### 3.1.1 Al_2_CoO_4_^+^

For this cation, the genetic algorithm^27^ yields the same isomers as previously reported for Al_2_FeO_4_^+^.^19^ The most stable isomers are a “cone”-like structure with a central trivalent O ion (C_s_-1) and a planar bicyclic frame with a terminal Al–O˙^−I^ radical and the transition metal in a M^+II^ oxidation state (C_s_-2). While the agreement between calculated and experimental spectra provides compelling evidence for the presence of C_s_-2, DFT with the TPSSh functional predicts C_s_-1 to be the lowest energy isomer.

#### 3.1.2 AlFe_2_O_4_^+^ and AlCo_2_O_4_^+^

For these cations, the global structure search produced a variety of structural isomers of which C_s_-1, C_s_-2, and C_1_-2 can be regarded as doubly transition metal-substituted analogs of the respective Al_2_MO_4_^+^ isomers. Additionally, there are isomers with two spiro-connected four-membered rings (D_2d_), a six-membered ring with a terminal Al–O˙^−I^ moiety (C_2v_), and a “key”-like structure which can be described as a four-membered ring attached to a nearly linear O–M^+III^–O^−II^ unit (C_1_-1). Fig. S6† provides bond distances and angles for C_1_-1 and C_1_-2.

For these isomers, none of the calculated harmonic IR spectra agrees as well with the corresponding IRPD spectra as it is the case for the C_s_-2 isomer of the Al_2_MO_4_^+^ cations, see Fig. 3. The predicted spectra of C_1_-2 and C_s_-2, the doubly substituted analogs of C_s_-2 for Al_2_MO_4_^+^, do not exhibit any resemblance to the experimental spectra. The D_2d_ and C_s_-1 isomers, which TPSSh predicts to be the lowest energy structures, do not provide good agreement between predicted and experimental vibrational spectra either.

The IRPD spectra of AlM_2_O_4_^+^ show a characteristic band just above 1050 cm^−1^ (Fe: c_1_; Co: c′_1_ in Table 1). It falls into the range in which the O–O stretching vibrations of superoxide (O_2_^−I^) moieties can be observed.^12^ However, such a unit is unlikely to be present in these systems as this would imply an oxidation state of M^+I^ for one of the transition metal ions, which is considered unfavorable for Fe and Co.

1 Al(M^+III^)_2_(O^−II^)_4_^+^ ⇔ Al(M^+I^)(M^+II^)(O_2_^–^^I^)(O^−II^)_2_^+^

Indeed, the most stable isomer with a superoxide moiety (C_s_-3) is 271 and 165 kJ mol^−1^ less stable than the TPSSh global minimum (D_2d_) for AlFe_2_O_4_^+^ and AlCo_2_O_4_^+^, respectively, see Fig. S10.† This is outside the expected TPSSh uncertainty for transition metal containing gas phase clusters of about 100 kJ mol^−1^.^19^ A superoxide-containing isomer is therefore discarded as a candidate for the experimentally observed structure. More importantly, for this isomer the overall agreement between predicted and experimental spectra is poor (see Fig. S10†).

The “key”-like C_1_-1 isomer is the only one without a superoxide unit which can explain the observed band above 1050 cm^−1^. It is assigned to an antiphase Al–O–M stretching vibration (Table 1, c_1_/c′_1_). In addition, for C_1_-1 two bands are predicted in the region of 600 to 850 cm^−1^ which are also experimentally observed (Table 1, c_3_/c′_3_ and c_4_/c′_4_). For a complete list of all vibrations of C_1_-1, see Table S6.† Therefore, the best agreement between the predicted and experimental vibrational spectra is obtained for the “key”-like isomer C_1_-1. Even though the experimental c_3_/c′_3_ and c_4_/c′_4_ bands exhibit substantially larger relative band intensities with respect to c_1_ than the predicted ones, there is no doubt that, if at all, it is the C_1_-1 isomer that gives rise to the observed IRPD spectrum.

### 3.2. IR spectra of AlFe_2_O_4_^+^ and AlCo_2_O_4_^+^ based on molecular dynamics simulations

To see if the experimental and predicted intensity ratios for the bands c_1_/c_3_ (c′_1_/c′_3_) and c_1_/c_4_ (c′_1_/c′_4_) for C_1_-1 can be improved by accounting for anharmonicity and finite temperature effects, we performed molecular dynamics (MD) simulations and determined the IR spectra by Fourier transformation of the dipole moments. To reach simulation times that are long enough to yield converged IR spectra with sufficient resolution, we employed machine learning interatomic potentials (MLIPs) derived from training data obtained from short DFT MD simulations (*ab initio* MD, AIMD). We have chosen this approach because directly performing MD simulations with forces and dipoles evaluated with hybrid exchange-correlation functionals such as TPSSh is computationally not feasible, see the Computational details section for further information.

**Fig. 4:**
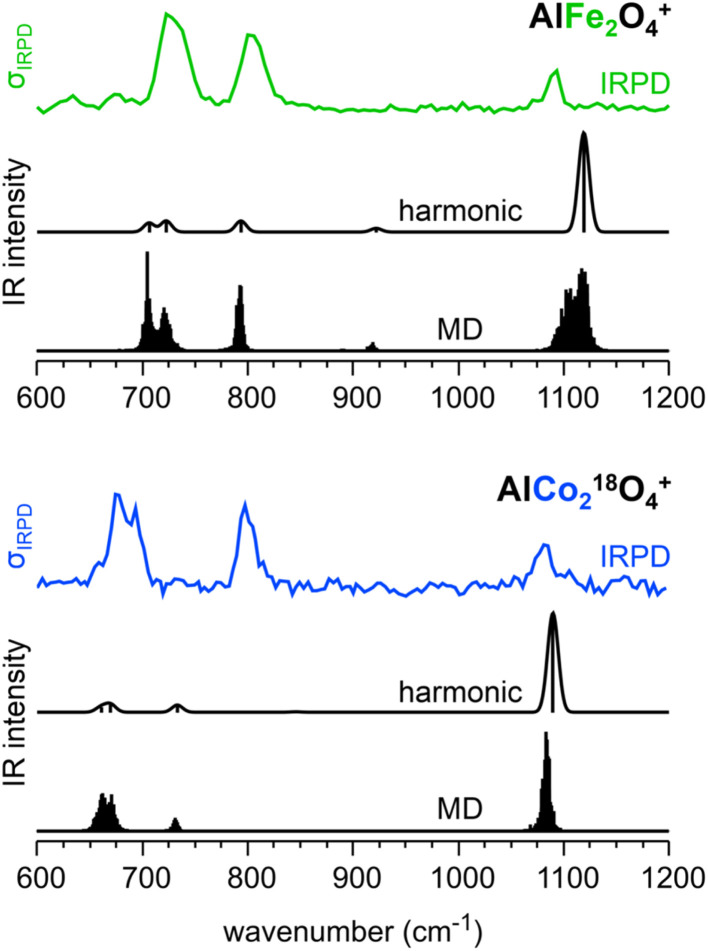
Experimental IRPD spectra of AlFe_2_O_4_^+^(He_1,2_) (green trace) and AlCo_2_^18^O_4_^+^(He_1,2_) (blue trace) as well as harmonic IR spectra (black traces) obtained with PBE0/def2-TZVPP and PBE0_MLIP_ MD (100 K) using machine learning interatomic potentials of the bare C_1_-1 cations.

Fig. 4 shows the IRPD spectra of C_1_-1 (“key”-like isomer) compared to the anharmonic IR spectra obtained from MD simulations and the harmonic spectra. Taking anharmonicities into account, the band positions remain similar, but the relative band intensities change substantially. The bands below 800 cm^−1^ gain in intensity relative to the band above 1050 cm^−1^, improving the agreement with the experimental IRPD spectra. We attribute the observed intensity differences between the harmonic and anharmonic IR spectra of C_1_-1 to the large amplitude motion of the O–M^+III^–O^−II^ unit, which results in a wide range of bending angles present in the MD simulations (see Section 5.4 of the ESI†). In contrast, the MD-based IR spectra of D_2d_ and C_s_-1 remain similar to the harmonic IR spectra in terms of band positions and relative band intensities (see Fig. S17†).

The IR spectra derived from the MD simulations improve the intensity ratios of c_1_/c_3_ (c′_1_/c′_3_) and c_1_/c_4_ (c′_1_/c′_4_) compared to the harmonic IR spectra (see Table S8†). Nevertheless, the intensity ratios are not the same as in the experiment and the c_1_ band of C_1_-1 in AlCo_2_O_4_^+^ remains the most intense. However, the improvement of intensity ratios provides additional support for the assignment of the observed IRPD spectra of AlFe_2_O_4_^+^ and AlCo_2_O_4_^+^ to the “key”-like C_1_-1 isomer.

### 3.3 Relative stability of isomers: dependence on the exchange-correlation functional

#### 3.3.1 Al_2_CoO_4_^+^

As previously reported for Al_2_FeO_4_^+^,^19^ the predicted stability of the C_s_-2 isomer relative to C_s_-1 strongly depends on the exchange-correlation functional employed in the DFT calculations (see Table 2). Functionals with less than 25% Fock exchange (also called exact exchange), such as TPSSh which we use here for structure and spectra predictions, yield C_s_-1 as the most stable isomer. Only functionals with a higher amount of Fock exchange predict C_s_-2 as the most stable isomer in agreement with the spectroscopic assignment. Whereas for Al_2_FeO_4_^+^, 50% Fock exchange was needed (BHLYP),^19^ for Al_2_CoO_4_^+^, the correct relative stability is obtained already with 25% (PBE0). It is known that the localization of an electron hole, *e.g.*, in the O-2p shell of oxide clusters (M^+II^/O˙^−I^ valence state),^19^ requires a minimum amount of Fock exchange in the functional which may vary from one system (Al_2_FeO_4_^+^) to another (Al_2_CoO_4_^+^).

**Table 2:** Relative stability in kJ mol^−1^ of the C_s_-2 relative to C_s_-1 isomers of Al_2_MO_4_^+^ (M = Fe, Co) obtained with different DFT functionals (% Fock exchange in parentheses)^a^ and different multi-reference wavefunctions methods

Method	Al_2_FeO_4_^+^^b^	Al_2_CoO_4_^+^
PBE (0)	93	74
TPSSh (10)	51	17
PBE0 (25)	27	−15
BHLYP (50)	−58	−127
MC-PDFT (tPBE)^c^	−72	24
MRCI-D	−56	−100

a High-spin states, see Tables S1–S3 for results obtained for different spin states.

b See Table 1 of ref. 19.

c Translated PBE functional.

Multireference configuration interaction calculations with Davidson corrections (MRCI-D) confirm that C_s_-2 is indeed the global minimum structure, which is 56 and 100 kJ mol^−1^ more stable than C_s_-1 for Al_2_FeO_4_^+^ (ref. 19) and Al_2_CoO_4_^+^, respectively, see Tables S2 and S3.† In contrast, the computationally much more affordable multi-configuration pair density functional theory (MC-PDFT),^32,33^ which uses the density from MC calculations in a translated PBE functional (tPBE),^34^ fails to give the right stability ordering for Al_2_CoO_4_^+^, whereas it gave the correct result for Al_2_FeO_4_^+^.^19^

#### 3.3.2 AlFe_2_O_4_^+^ and AlCo_2_O_4_^+^

Comparison of the TPSSh predictions for the IR spectra and the collision cross sections leaves no doubt that it is the “key”-like C_1_-1 isomer that is observed in the IRPD and ion mobility experiments. However, it is not predicted to be the global minimum structure, neither with the TPSSh functional, nor with any of the other functionals we have tested (see Table 3). The PBE, TPSSh, PBE0 and BHLYP functionals predict C_1_-1 to be 96 to 101 kJ mol^−1^ (AlFe_2_O_4_^+^) and 37 to 50 kJ mol^−1^ (AlCo_2_O_4_^+^) less stable than the respective global minimum structures.

**Table 3:** Relative stabilities in kJ mol^−1^ of different isomers of AlFe_2_O_4_^+^ and AlCo_2_O_4_^+^ as obtained for the high spin states with different functionals (% Fock exchange in parentheses)

Isomer	AlFe_2_O_4_^+^				AlCo_2_O_4_^+^			
	PBE (0)	TPSSh (10)	PBE0 (25)	BHLYP (50)	PBE (0)	TPSSh (10)	PBE0 (25)	BHLYP (50)
D_2d_	0	0	10	51	0	0	0	83
C_s_-1	16	1	0	12	28	14	13	101
C_2v_	122	69	60	0	53	20	10	0
C_1_-1	**96**	**97**	**101**	**97**	**47**	**41**	**37**	**50**
C_1_-2	—a	101	78	6	—a	53	22	4
C_s_-2	156	130	109	42	95	95	68	61

a Converts into C_2v_.

So far, we have assumed that both transition metal ions are in a high spin state and ferromagnetically coupled. The transition metal and O ions of D_2d_, C_s_-1, and C_1_-1 are all in a +III and –II oxidation state, respectively (see Fig. S7†). When lower spin states at the transition metal ions or antiferromagnetic coupling are considered, the stability order does not change and D_2d_ remains the global minimum structure with TPSSh (see Tables S4 and S5†). Furthermore, the agreement of the predicted IR spectra of C_1_-1 with the experimental IRPD spectra does not improve when considering lower spin states (see Fig. S12†).

In summary, none of the tested DFT functionals is able to correctly predict the relative stability of different AlFe_2_O_4_^+^ and AlCo_2_O_4_^+^ isomers.

### 3.4. Collision cross sections

Since the “key”-like C_1_-1 isomer is structurally so different from the compact “spiro” D_2d_ found to be most stable with TPSSh and also from other low-energy structures such as C_s_-1, we provide additional evidence for the presence of the C_1_-1 structure from ion mobility measurements (see Section 2.4).^15,16^Table 4 compares the experimental and theoretical CCSs of each isomer of the AlM_2_O_4_^+^ cations. The “key”-like C_1_-1 structures (71.4 and 72.5 Å^2^ for M = Fe and Co, respectively) are unequivocally assigned as the experimentally observed structures. The deviations between the experimental and theoretical CCSs and arrival times are within the experimental uncertainty limits. The second, less intense peak in the arrival time distribution for AlFe_2_O_4_^+^ (Fig. 2, 67.0 ± 0.9 Å^2^) is assigned to the planar bicyclic structure-type C_s_-2 or C_1_-2 structure. The CCS deviation between prediction and experiment (−2.5 ± 0.9 Å^2^) is larger, but this assignment is supported by the results for the mono-substituted Al_2_FeO_4_^+^ cation. Consistent with the original assignment based on the IRPD and MRCI data,^16^ the best agreement between experiment and theory for the CCS is obtained for the planar bicyclic structure C_s_-2. For Al_2_FeO_4_^+^, the deviation is also −2.5 ± 1.1 Å^2^. The intensity ratio between the 70.6 and 67.0 Å^2^ peaks for AlFe_2_O_4_^+^, assigned to the C_1_-1 (“key”) and C_s_-2 (“ladder”) isomers, is 1 : 0.3.

**Table 4:** Calculated collision cross sections (CCSs) in A^2^ and expected arrival time (*t* in µs) of different AlFe_2_O_4_^+^ and AlCo_2_O_4_^+^ isomers, calculated at *T*_eff_ = 122 K (see Methods section for further information). Experimental CCSs and arrival times are also shown. Differences between experiment and predictions are given in parenthesis

Al_2_FeO_4_^+^		AlFe_2_O_4_^+^				AlCo_2_O_4_^+^	
Isomer	CCS	Type	Isomer	CCS	*t*	CCS	*t*
		Spiro	D_2d_	63.6	289	67.4	308
C_s_-1	61.8	Cone	C_s_-1	59.4	275	64.3	297
			C_2v_	63.7	290	67.9	310
		Key	C_1_-1	71.4	316	72.5	326
C_s_-2	65.9	Ladder	C_1_-2	64.5	292	68.6	312
		Ladder	C_s_-2	64.8	293	68.5	312
Exp	68.4 ± 1.1		Exp	70.6 ± 0.9	312 ± 3	71.4 ± 0.8	322 ± 3
(C_s_-2)	(−2.5 ± 1.1)		(C_1_-1)	(0.8 ± 0.9)	(4 ± 3)	(1.1 ± 0.9)	(4 ± 3)
			Exp	67.0 ± 0.9	298 ± 3		
			(C_1_-2)	(−2.5 ± 0.9)	(−6 ± 3)		
			(C_s_-2)	(−2.2 ± 0.9)	(−5 ± 3)		

### 3.5. Reactivity calculations

Whereas Al_3_O_4_^+^ exhibits a “cone”-like *C*_3v_ structure,^17^ substitution with an Fe^3+^ ion results in a structural rearrangement, forming a planar bicyclic “ladder”-like *C*_s_ structure for Al_2_FeO_4_^+^ (C_s_-2 in Fig. 3) with a highly reactive terminal Al–O˙^−I^ oxygen radical site.^22,35^ This is accompanied by the change from the Fe^+III^/O^−II^ to the Fe^+II^/O˙^−I^ valence state which is also observed for Co^+III^ in Al_2_CoO_4_^+^ (see Fig. 3) and for Ni^+III^ in Al_2_NiO_4_^+^(see ref. 21). The experiments conducted in this work confirm that Al_2_FeO_4_^+^ and Al_2_CoO_4_^+^ readily react with methane (see Fig. S2†).

In agreement with the experimental observation that AlFe_2_O_4_^+^ and AlCo_2_O_4_^+^ exhibit no signs of reactivity towards methane (see Fig. S2†) our TPSSh calculations for the “key”-like C_1_-1 isomer yield positive apparent energy barriers Δ*E*_0_ of 44 and 29 kJ mol^−1^, respectively, for the C–H bond activation (see Fig. S18†). Methane initially attaches to the two-fold coordinated transition metal ion in the four-membered ring with binding energies of −75 and −86 kJ mol^−1^ for AlFe_2_O_4_^+^ and AlCo_2_O_4_^+^, respectively. Given that all transition metal and O ions in C_1_-1 are in a +III and –II oxidation state, respectively, H atom abstraction proceeds *via* proton coupled electron transfer, M^+III^(d^*n*^)/O^2−^ + H˙ → M^+II^(d^*n*+1^)/OH^−^, rather than *via* H atom transfer as found for the planar bicyclic C_s_-2 structure of Al_2_FeO_4_^+^, M^+II^(d^*n*^)/O˙^−^ + H^•^ → M^+II^(d^*n*^)/OH^−^. However, due to the positive apparent barrier, methane would dissociate again from the cluster before any type of C–H bond activation could occur.

## 4 Discussion and conclusions

**Fig. 5:**
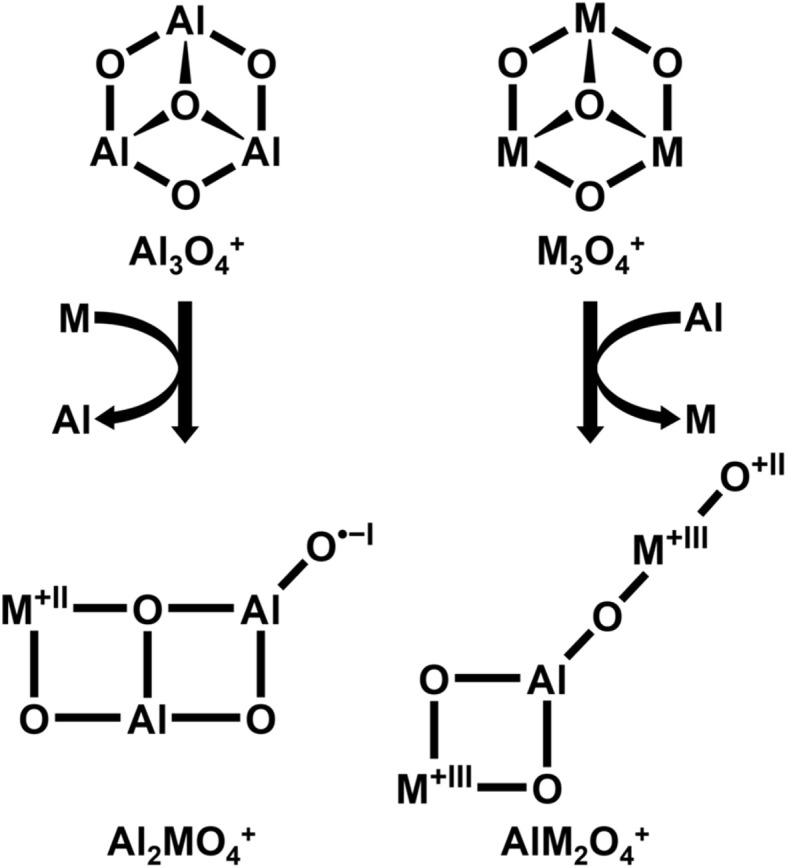
Schematic representation of the change in structure and valence upon composition change from Al_3_O_4_^+^ to Al_2_MO_4_^+^ and M_3_O_4_^+^ to AlM_2_O_4_^+^ with M = Fe, Co.

All three metal oxide clusters, Al_3_O_4_^+^,^17^ Fe_3_O_4_^+^,^26^ and presumably Co_3_O_4_^+^ (ESI, Section S3†) share the same “cone”-like *C*_3v_ structure which has a threefold coordinated O ion on top of a six-membered (MO)_3_ ring. Fig. 5 summarizes the structural changes caused by substitution of an Al ion for a transition metal (M = Fe, Co) in M_3_O_4_^+^ yielding AlM_2_O_4_^+^, and by substitution of a transition metal ion for Al in Al_3_O_4_^+^ yielding Al_2_MO_4_^+^. For both substitutions, the observed IR spectra (Fig. 1) indicate a significant structural change compared to the respective parent cluster, but no structural differences between Fe- and Co-containing clusters of the same composition.

Substitution of M = Fe^19^ and Co (this work) for Al in Al_3_O_4_^+^ changes the “cone”-like *C*_3v_ structure into a planar bicyclic structure (C_s_-2 in Fig. 3) which is accompanied by a change of the valence state from M^+III^/O^−II^ to M^+II^/O˙^−I^. The unpaired electron at the terminal oxygen site explains the observed hydrogen abstraction from methane. In contrast, the Al substitution in M_3_O_4_^+^ (M = Fe, Co) changes the “cone”-like *C*_3v_ structure of M_3_O_4_^+^ to a different, “key”-like *C*_1_ structure of AlM_2_O_4_^+^ (C_1_-1 in Fig. 3) which does not show reactivity towards methane. It consists of a four-membered ring connected *via* a central three-fold coordinated Al^3+^ ion to a nearly linear O^−II^–M^+III^–O^−II^ unit.

Comparison of the IRPD spectra and IM-MS data together with the computational predictions for the “key”-like C_1_-1 structure leaves no doubt that this is the experimentally observed AlM_2_O_4_^+^ isomer. It is the only isomer which explains the observed characteristic band above 1050 cm^−1^. But, according to the DFT calculations, C_1_-1 is not the global minimum energy structure. For AlFe_2_O_4_^+^ and AlCo_2_O_4_^+^, it is 96 to 101 and 37 to 50 kJ mol^−1^, respectively, less stable than the respective global minimum structures. Such failures of DFT with transition metal compounds are not unexpected. Although TPSSh does not yield C_1_-1 as the global minimum structure, it still provides good agreement with the CCSs and IRPD band positions. This illustrates that the different functionals may accurately describe the local minimum structures and the shape of the PES around them, but not the relative energies of isomers with differently coordinated transition metal ions.

Reliable results for transition metal oxides require multi-reference wavefunction calculations. Whereas for the Al_2_MO_4_^+^ ions MRCI-D calculations could be completed, see ref. 19 and Section 3.3, for AlM_2_O_4_^+^, the presence of two transition metal ions would require larger active spaces for which such calculations cannot be converged with the currently available methods. However, we may learn something about the differences between TPSSh and MRCI-D results from available calculations on related, even smaller gas phase systems. FeO_2_^−^ is a subunit in both the “spiro” D_2d_ isomer (most stable with TPSSh) and the “key”-like C_1_-1 isomer (best match with experiment) of AlM_2_O_4_^+^. Previously, Müller found that FeO_2_^−^ ions prefer a bent structure according to TPSSh, but a linear one according to MRCI.^36^ The “spiro” D_2d_ structure has two bend FeO_2_^−^ subunits connected by the central Al ion. If one of them opens and becomes linear the “key”-like C_1_-1 isomer is obtained. With MRCI this transition would be connected with an energy gain making the C_1_-1 structure more stable than the “spiro” D_2d_ structure. This consideration could be made more quantitative with calculations using the localized active space (LAS) approach, which defines active spaces separately on connected sub-units.^37^

In conclusion, isovalent metal substitution in metal oxides induces structural changes that are much larger for gas phase clusters than for the corresponding bulk oxides. Starting from cone-shaped M_3_O_4_^+^ structures in which all metal ions are equal (the same M^+III^ oxidation state and the same three-fold coordination with oxygen ions) and in the absence of periodic constraints, the substituted clusters (M = Fe, Co) attain more stable structures of different type in which the metal ions are in different oxidation states (M^+II^ in Al_2_MO_4_^+^, “ladder”) or have different coordination numbers (two-fold coordination in AlM_2_O_4_^+^, “key”-structure).

## 5 Experimental methods

### 5.1. Infrared photodissociation spectroscopy

The infrared photodissociation (IRPD) experiments were performed employing a cryogenic ion trap tandem mass spectrometer^38,39^ using the widely tunable, intense IR radiation from the Fritz-Haber-Institute Free-Electron-Laser (FHI FEL).^40^ The gas phase ions were generated in a pulsed laser vaporization source by focusing a frequency-doubled Nd:YAG laser (50 Hz, 10–15 mJ) onto a rotating mixed metal rod (Al/TM of 70/30 and 18/82 at% for the mono and doubly substituted clusters, respectively). The resulting plasma was quenched with a gas pulse of 0.5% O_2_ seeded in He. Throughout all experiments, the Oxygen-18 isotope was used with the Co/Al target to reduce the number of isobars formed by avoiding clusters containing Al^16^O_2_ moieties, which are isobaric with Co (59 u). Cluster ions were formed during expansion through a clustering channel downstream from the rod and passed through a 4 mm diameter skimmer. The beam of ions was then collimated and thermalized close to room temperature in a He-gas filled radio frequency (RF) ion guide, mass-selected using a quadrupole mass-filter, and focused into a cryogenically cooled RF ring-electron ion-trap. The trap was continuously filled with He-gas at a trap temperature of 11 to 15 K or with a reactant gas/buffer gas mix of 0.01% CH_4_ in He. Many collisions of the trapped ions with the gas particles provided gentle cooling of the internal degrees of freedom close to the ambient temperature. Under these conditions, the He-tagged species are formed by three-body collisions.^23^ All ions are extracted from the ion trap and focused both temporally and spatially into the center of the extraction region of an orthogonally mounted reflectron time-of-flight (TOF) tandem mass spectrometer. Here, the ions are irradiated with a single counterpropagating IR laser macropulse (duration: 10 µs) produced by the FHI FEL (430–1200 cm^−1^, 5 Hz), with a bandwidth of ∼0.5% fwhm and pulse energy of 0.7–2.5 mJ. Parent as well as photofragment ion yields are monitored simultaneously as a function of the irradiation wavenumber. IRPD scans are recorded by averaging over 100 TOF mass spectra per wavenumber step (2 cm^−1^). Typically, at least three scans are summed to obtain the final IRPD spectrum. The photodissociation cross section *σ*_IRPD_ is determined as described previously.^14,41^

### 5.2. Ion mobility-mass spectrometry

Ion mobility-mass spectrometry was performed using a home-built vacuum apparatus composed of a cluster ion source, an ion drift tube, and a time-of-flight mass spectrometer. Details of experiments were already reported elsewhere.^16^ Fe/Al alloy oxide cluster cations were generated by a combination of laser vaporization of a Fe/Al alloy rod and supersonic expansion of 5% O_2_/He mixture gas (stagnation pressure = 0.3 MPa). For formation of Co/Al alloy oxide cluster cations, we used double-rod type laser vaporization source where Co and Al rods were vaporized by two YAG lasers independently to reduce the number of isobars possible. In the present experiment, the power of laser for vaporization of a Co rod (5.7 mJ per pulse) was slightly higher than that of an Al rod (3.6 mJ per pulse). With this condition, we confirmed that the contribution of CoAl_2_O_6_^+^ can be negligible in the arrival time distribution of Co_2_AlO_4_^+^. The generated ions were injected into the drift tube with a kinetic energy of 50 eV by a pulsed electric field at a given time (*t* = *t*_0_). The drift tube was 107 mm long and was filled with He buffer gas with a pressure of 1.00 Torr at 100 K. The drift electric field in the tube was *E* = 11.2 V cm^−1^. The *E*/*N* value was 11.5 Td (*N* is the number density of the buffer gas, 1 Td = 10^−17^ V cm^2^). After running through the drift tube, the ions were reaccelerated to ∼1.8 keV by another pulsed electric fields in an acceleration region of the time-of-flight mass spectrometer at a given time later from the first pulse: *t* = *t*_0_ + Δ*t*. The ions were introduced to the mass spectrometer and detected by a dual microchannel plate.

The delay time between the two pulses, Δ*t*, was defined as “arrival time”. The drift velocity of the ions in the drift tube, *v*_d_, was calculated numerically to satisfy the measured arrival time. We also obtained the time that an ion spends in the drift tube, *t*_d_, from the measured arrival time. It is known that *v*_d_ is proportional to *E*, and the proportional constant (*K*) is called as ion mobility. The mobility *K* depends on the number density of the buffer gas *N*. To compare the mobility under different experimental conditions, the reduced mobility *K*_0_ is defined as *K*_0_ = *K*·(*N*/*N*_0_), where *N*_0_ is Loschmidt's number. From the Mason–Schamp equation, the reduced mobility *K*_0_ in the drift tube was given as2
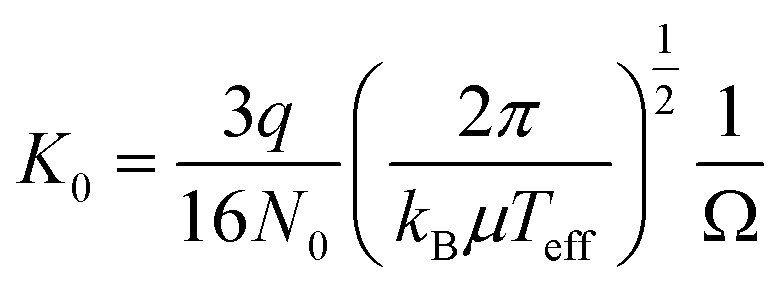
where *q* is the charge of the ion, *k*_B_ is the Boltzmann constant, *µ* is the reduced mass of the ion and the buffer gas atom, *T*_eff_ is the effective temperature of the ions, and *Ω* is a collision cross section. The effective temperature is given by *T*_eff_ = *T*_BG_ + *m*_BG_*v*^2^_d_/3 *k*_B_, where *T*_BG_ is the buffer gas temperature, and *m*_BG_ is the mass of buffer gas. In the present experimental condition, *T*_eff_ was about 122 K for AlCo_2_O_4_^+^ and AlFe_2_O_4_^+^. The collision cross section of the ion is calculated from the measured arrival time of the ion, Δ*t*.

Theoretical collision cross sections (CCS_calc_) were calculated by using the trajectory method in the MOBCAL program. We used the parameters of Lennard-Jones potentials in the trajectory calculations. These parameters were determined to reproduce the experimental collision cross sections (CCS_exp_) of cobalt, iron, and aluminum oxide cluster ions.

### 5.3. Computational methods

#### 5.3.1 Density functional theory

The energetically most stable structures of Al_2_CoO_4_^+^, AlFe_2_O_4_^+^, and AlCo_2_O_4_^+^ were identified using a genetic algorithm (GA)^27,28^ with the BP86,^42,43^ PBE0,^44^ and BHLYP^45^ exchange correlation functionals and the def2-SVP^46^ basis set for the respective high spin states, assuming trivalent transition metal ions. Each GA generation comprised 30 structures, and 40 different generations were generated, yielding a total of 1200 structures per functional for each composition. The GAs were considered converged when the energy of the most stable isomer remained unchanged. The structures of the most stable unique isomers were reoptimized with the functionals PBE,^47,48^ TPSSh,^29^ PBE0,^44^ and BHLYP^45^ using the def2-TZVPP^30,31^ basis set and the m5 integration grid as implemented in Turbomole V7.2.^49–52^ Starting from the high spin structures and wavefunctions, lower spin states were optimized using the same settings. Harmonic frequencies and vibrational normal modes were obtained using the double harmonic approximation with the TPSSh^29^ functional and def2-TZVPP^30,31^ basis set. The line plots of the harmonic IR spectra were obtained as a convolution of calculated frequencies and intensities with a Gaussian line-shape function with a 10 cm^−1^ width at half-maximum.

#### 5.3.2 Multireference calculations

Multiconfigurational self-consistent field single point calculations were conducted using TPSSh/def2-TZVPP optimized structures with the complete active space (CAS) formalism. The CAS(X,Y) nomenclature indicates the number of active electrons and orbitals, respectively. The active spaces were constructed to always include the TM 3d orbitals and zero to five O 2p orbitals, depending on the CAS size. Furthermore, an all-valence active space was used, including all 2p orbitals of all O-ions in addition to the TM 3d orbitals. The TM 1s2s2p3s3p, O 1s2s, and Al 1s2s2p orbitals were kept frozen during the single point calculations. Dynamic correlation effects were incorporated through the (i) multireference configuration interaction with singles and doubles (MRCISD or MRCI)^53,54^ with additional size-consistency corrections as suggested by Davidson (MRCI-D)^55^ and Pople (MRCI-P)^56^ as well as (ii) the perturbative approaches complete active space perturbation theory (CASPT2)^57^ and N-electron valence state perturbation theory (NEVPT2).^58–60^

All multireference calculations were performed using the correlation consistent polarized core valence triple-ζ (cc-pwCVTZ)^61–64^ basis sets. CASSCF, CASPT2, NEVPT2, and MRCI calculations were performed with the Molpro program package V2015.1,^65^ and multiconfiguration pair-density functional theory (MC-PDFT)^32,33^ calculations with the translated PBE functional (tPBE) using the openMOLCAS program V18.0.^66–68^

#### 5.3.3 Machine learning interatomic potentials

Fully anharmonic, finite temperature IR spectra were obtained using DFT quality MD simulations based on MLIPs. As simulation times of up to 500 ps are required, see Fig. S16,† which equals one million energy/force evaluations with the chosen settings, the direct *ab initio* approach is computationally not feasible. A single energy/force evaluation with the *ab initio* approach requires 270 s CPU execution time on an Intel Xeon “Haswell” processor E5-2667 v3, whereas the MLIP approach takes only 72 ms GPU execution time on a NVIDIA V100 GPU. This equals a 3750-fold speed up compared to the *ab initio* approach.

We build on the MACE architecture,^69^ as it allows for fast and highly data-efficient training with high-order equivariant message passing and has been successfully used to generate IR spectra in other contexts.^70–72^ To represent the PES and dipole moment surface (DMS), we train MLIPs to both (i) energies and forces, and (ii) dipole moments. For each isomer, independent training data sets are generated. For this, DFT MD simulations were run in a canonical NVT ensemble using the Nose–Hoover thermostat at a simulation temperature of 600 K and a time step of 0.5 fs for a total simulation time of 16 ps with the PBE0 (ref. 44) exchange correlation functional and the def2-TZVPP^30,31^ basis set. A total of 1600 structures were randomly selected from four independent DFT MD runs, the structures were shifted to the coordinate system origin, aligned by minimizing their RMSD, and the energy, gradient and dipole moment were recalculated with the same computational method. To accurately reproduce the PES, we used MACE models with two layers, a spherical expansion up to *l*_max_ = 3, a cutoff distance of 5 Å, and 128 equivariant messages. To reproduce the DMS, we used MACE models with 32 channels, a spherical expansion up to *l*_max_ = 2, and a cutoff distance of 5 Å.

The MLIPs reproduce the underlying DFT data accurately (see Fig. S13†). The MDs are run by the Atomic Simulation Environment (ASE)^73^ using the MLIPs trained to reproduce the PESs. We conducted the MLIP MD simulations at a simulation temperature of 100 K and simulation time of 250 ps for D_2d_ and C_s_-1 as well as 500 ps for C_1_-1 with a 0.5 fs time step using a Langevin thermostat with a friction coefficient of 5 ps^−1^. The longer simulation time for C_1_-1 was chosen to provide fully converged MD-based IR spectra (Fig. S16†). We then evaluated the dipole moments for the structures of the MD trajectories using the MLIPs trained to reproduce the DMSs. MD based IR spectra were subsequently generated by Fourier-transforming the dipole moments.

## Data availability

The authors confirm that the data supporting the findings of this study are available within the article and its ESI.†

## Author contributions

W. Schwedland: conceptualization, data curation, formal analysis, investigation, methodology, project administration, validation, visualization, writing – original draft, writing – review & editing. T. Penna: conceptualization, data curation, formal analysis, investigation, project administration, validation, visualization, writing – review & editing. H. Windeck: data curation, formal analysis, investigation, writing – original draft, writing – review & editing. F. Müller: data curation, investigation, methodology, supervision. S. Leach: data curation, investigation. J. Sauer: conceptualization, funding acquisition, project administration, resources, supervision, writing – original draft, writing – review & editing. X. R. Advincula: data curation, investigation, software. F. Berger: conceptualization, formal analysis, investigation, methodology, project administration, supervision, writing – original draft, writing – review & editing. N. Ishida: data curation, formal analysis, investigation, validation. K. Ohshimo: data curation, formal analysis, investigation, writing – original draft, writing – review & editing. F. Misaizu: conceptualization, funding acquisition, project administration, resources, supervision, writing – original draft, writing – review & editing. Y. Li: conceptualization, project administration, data curation, formal analysis, investigation, validation A. Chakraborty: conceptualization, project administration, data curation, formal analysis, investigation, validation F. Horn: data curation, formal analysis, investigation, validation. K. R. Asmis: conceptualization, funding acquisition, project administration, resources, supervision, writing – original draft, writing – review & editing.

## Conflicts of interest

There are no conflicts to declare.

## Supplementary Material

SC-OLF-D5SC02681D-s001

SC-OLF-D5SC02681D-s002
