# Supplementary material for: Unexpected structural isomers of AlFe2O4+ and AlCo2O4+: vibrational spectroscopy and ion mobility combined with quantum chemistry
Source: Chem Sci. 2025 May 19;16(26):11999–2011. doi: 10.1039/d5sc02681d (PMC12134933; doi:10.1039/d5sc02681d)
Supplement: SC-016-D5SC02681D-s002 [file SC-016-D5SC02681D-s002.pdf]

Supplementary Information (SI) for Chemical Science.
This journal is © The Royal Society of Chemistry 2025

Electronic Supplementary Information

Unexpected Structural Isomers of $AlFe_2O_4^+$ and $AlCo_2O_4^+$: Vibrational Spectroscopy and Ion Mobility combined with Quantum Chemistry

Winni Schwedland[a+], Tatiana C. Penna[c,d+], Henning Windeck[a], Fabian Müller[a], Stephen Leach[a], Joachim Sauer[a*], Xavier R. Advincula[b], Fabian Berger[a,b*], Nanako Ishida[e], Keijiro Ohshimo[e], Fuminori Misaizu,[e*], Ya-Ke Li[c,d], Arghya Chakraborty[c,d], Francine Horn[c,d], and Knut R. Asmis[c*]

[a] Institut für Chemie, Humboldt-Universität zu Berlin, Unter den Linden 6, 10099 Berlin (Germany), E-Mail: js@chemie.hu-berlin.de

[b] Yusuf Hamied Department of Chemistry, University of Cambridge, Lensfield Rd, Cambridge CB2 1EW (United Kingdom), E-mail: fb593@cam.ac.uk

[c] Wilhelm-Ostwald Institut für Physikalische und Theoretische Chemie, Universität Leipzig, Linnéstraße 2, 04103 Leipzig (Germany), E-Mail: knut.asmis@uni-leipzig.de

[d] Fritz-Haber-Institut der Max-Planck-Gesellschaft, Faradayweg, 4-6, 14195 Berlin, Germany.

[e] Graduate School of Science, Tohoku University, 6-3 Aoba, Aramaki, Aoba-ku, Sendai 980-8578. Japan, E-Mail: misaizu@tohoku.ac.jp

[+] These authors contributed equally to this work.

Table of Contents

S2. Ion Mobility Mass Spectrometry – Additional Results 5

S3. Additional Computational Results for $Al_3O_4^+$ and $Fe_3O_4^+$ 5

S4. Additional Computational Results for $Al_2CoO_4^+$ 6

S4.1. Density Functional Theory – Relative Stability 6

S4.2. Multireference Calculations – Relative Stability 7

S5. Additional Computational Results for $AlFe_2O_4^+$ and $AlCo_2O_4^+$ 8

S5.1. Density Functional Theory – Geometric and Electronic Structure 8

S5.2. Density Functional Theory – Relative Stability 9

S5.3. Density Functional Theory – Harmonic Vibrational Spectra 10

S5.4. Density Functional Theory – Vibrational Spectra by Molecular Dynamics 13

S5.5. Density Functional Theory – Reactivity Calculations 17

References 18

S1. Mass Spectrometry and IRPD – Additional Results

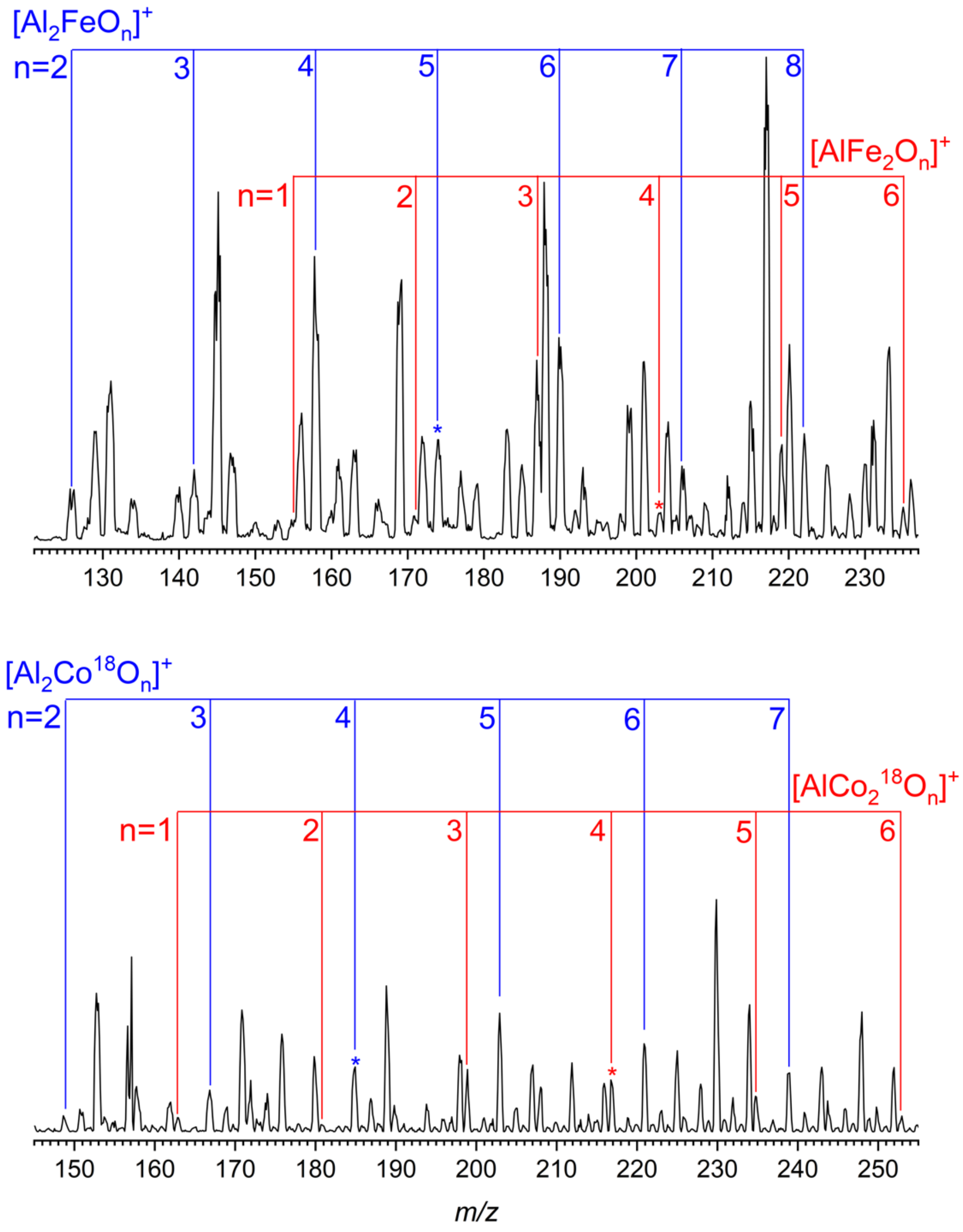

Fig. S1 Quadrupole mass spectra of the cations produced by the laser ablation source of (top) Fe/Al 80/20 rod and (bottom) Co/Al 82/18 rod in %, with 0,5% seeded O_2 in Helium (for Co, ^{18}O was used). The main series of the clusters of interest are highlighted.

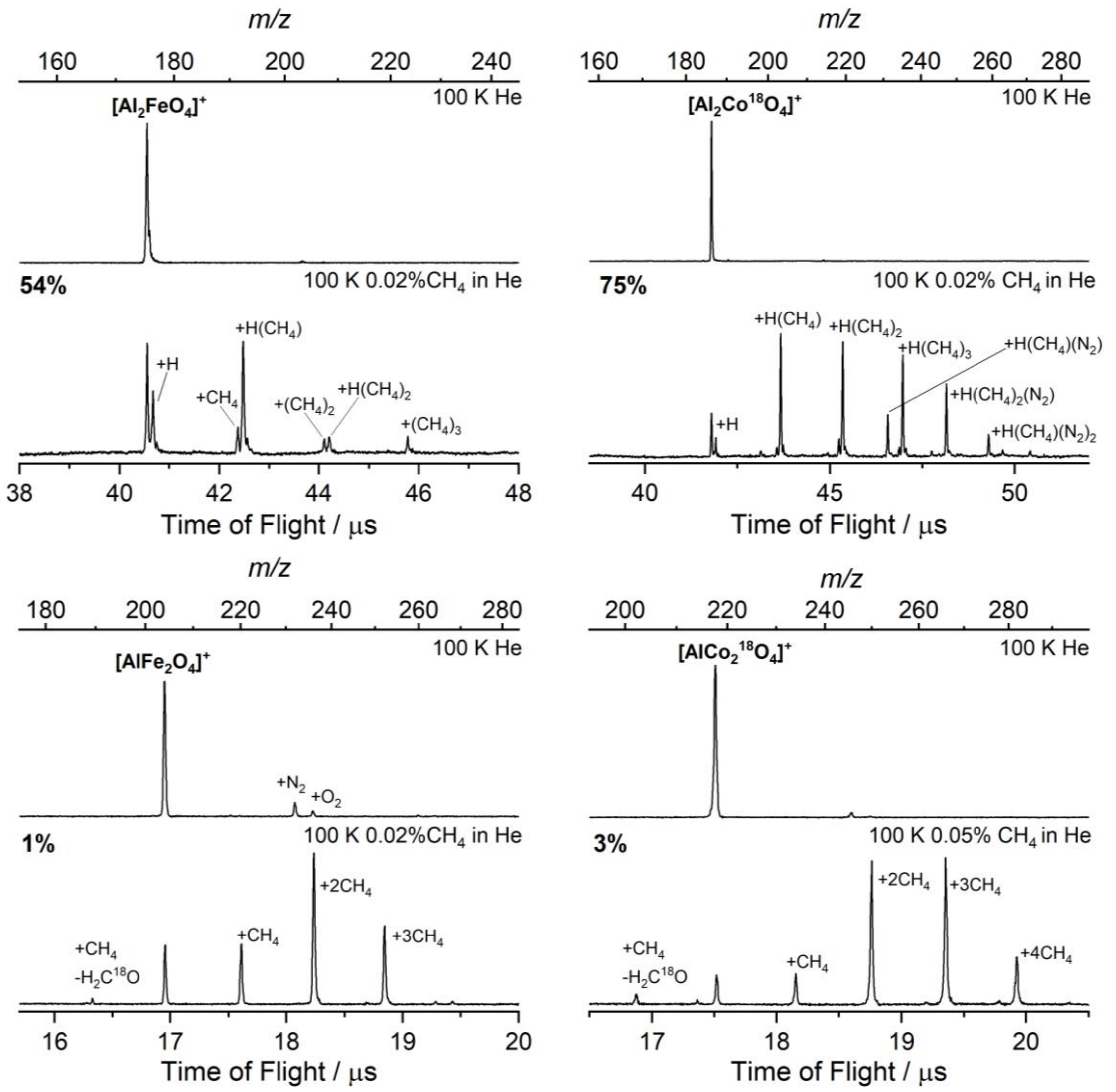

Fig. S2 Time-of-flight mass spectra obtained after storing mass selected cations top up to 200 ms in the ion trap filled with He, or CH_4 seeded in He at 100 K. The reactant ions and products are labeled for each panel. The reaction efficiency, calculated as the ratio between the peak area of products over total peak area is show for each system in %.

S2. Ion Mobility Mass Spectrometry – Additional Results

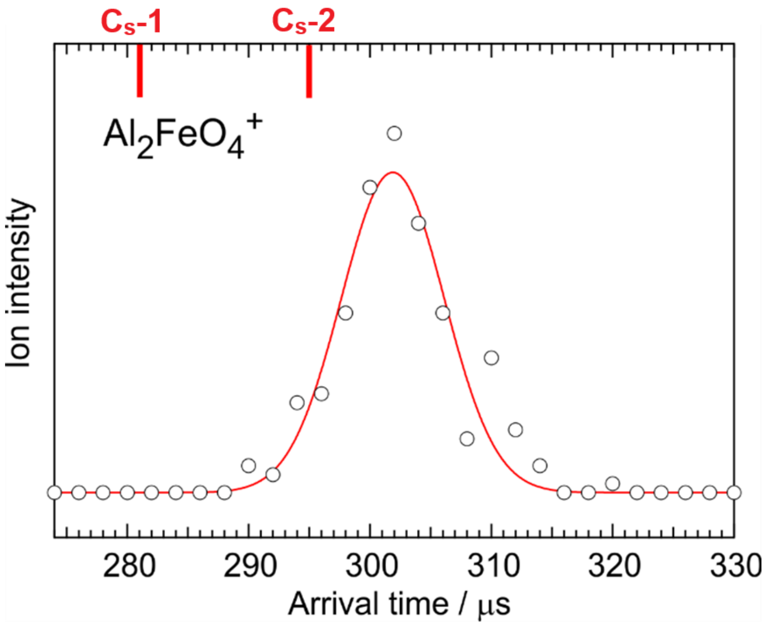

Fig. S3. The arrival time distribution of $Al_2FeO_4^+$. A red solid curve is a Gaussian function which is used for fitting the experimental plots (black circles). The width of the Gaussian function was determined from the experimental resolution of our IM-MS apparatus. The pressure and temperature of buffer He gas were 0.993 Torr and 98.9K, respectively. Red vertical bars indicate the arrival time predicted from the theoretical collision cross sections of each isomer.

S3. Additional Computational Results for $Al_3O_4^+$ and $Fe_3O_4^+$

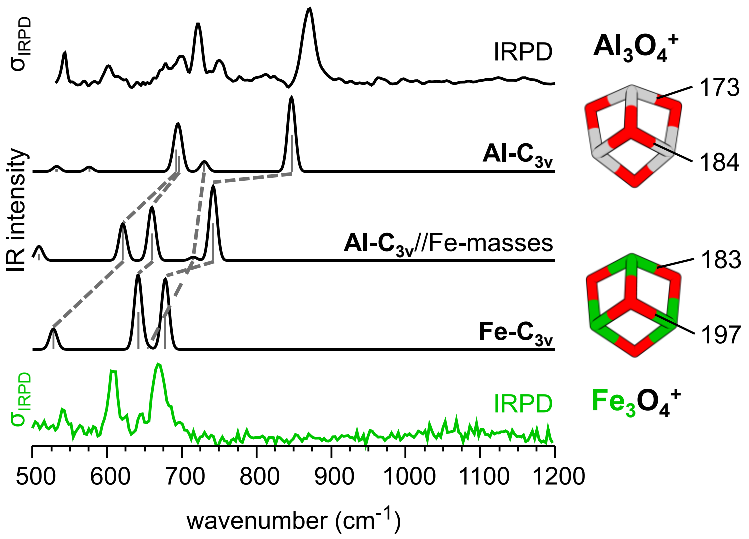

Fig. S4 IRPD spectra of $Al_3O_4^+(He_3)^+$,[17] and $Fe_3O_4(He)^+$,[22] with the corresponding harmonic IR spectra of the bare cations **C_{3v}** as calculated with TPSSh/def2-TZVPP for the high spin states. Bond lengths are given in pm and Al-C_{3v}//Fe-masses denotes the structure of Al-C_{3v} with the metal atom mass of Fe. Dashed lines correspond to same vibrational normal modes.

S4. Additional Computational Results for $Al_2CoO_4^+$

S4.1. Density Functional Theory – Relative Stability

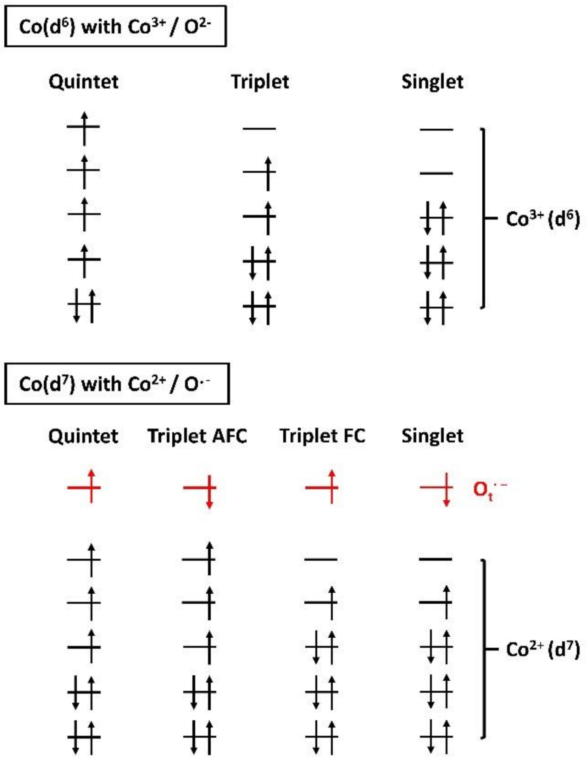

Fig. S5 Considered spin states of $Al_2CoO_4^+$ **C_s-1** and **C_s-2**. The black bars resemble the five *d*-orbitals, the red bars the singly occupied *p*-orbital at the terminal oxygen ions (O^t), and the arrows alpha and beta electrons, respectively. For the isomers following the Co^{+III}/O^{-II} motif in their electronic structure, both triplet and singlet state are established by pairing of *d*-electrons towards local low spin states at the cobalt atom. For the isomers with the $Co^{+II}/O^{\cdot-I}$ motif, lowering of the spin is either achieved by antiferromagnetic coupling (AFC) with O^t or by ferromagnetic coupling (FC) with pairing of *d*-electrons.

Table S1 Relative stability of $Al_2CoO_4^+$ **C_s-1** and **C_s-2** in kJ mol^{-1} in different spin states (2S+1) with respect to the **C_s-2** high spin state as obtained with different DFT-functionals (% Fock exchange in parentheses) and the def2-TZVPP basis set.

Isomer	2S+1	PBE (0)	TPSSh (10)	PBE0 (25)	BHLYP (50)
**C_s-1**	**5**	−74	−17	15	127
	**3**	−61	4	57	113
	**1**	−48	31	99	253
**C_s-2**	**5**	0	0	0	0
	**3** (AFC)[a]	1	0	−1	0
	**3** (FC)[b]	44	58	71	97
	**1**	45	58	71	97

[a]Antiferromagnetic coupling [b]Ferromagnetic coupling according to Fig. S5

S4.2. Multireference Calculations – Relative Stability

Table S2 Relative energies of $Al_2MO_4^+$ (M = Fe, Co) **C$_s$-1** and **C$_s$-2** in kJ mol^{-1} in different spin states (2S+1) as obtained with different multi-reference methods. Active space sizes are (11,11) for $Al_2FeO_4^+$ and (10,10) for $Al_2CoO_4^+$. Methodology analogous to ref. [1].

Isomer	2S+1		MC-PDFT		NEVPT2		MRCI-D		MRCI-P	
	Fe	Co	Fe	Co	Fe	Co	Fe	Co	Fe	Co
**C$_s$-1**	**6**	**5**	72	−24	83	0	56	100	48	85
	**4**	**3**	104	10	157	91	166	167	155	153
	**2**	**1**	46	−20	149	140	-	176	-	159
**C$_s$-2**	**6**	**5**	0	0	0	0	0	0	0	0
	**4**	**3**	0	0	0	0	0	0	−2	−2
	**2**	**1**	113	69	129	116	-	140	-	133

Table S3. Relative energies of $Al_2(M)O_4^+$ (TM = Fe, Co) **C$_s$-1** with respect to **C$_s$-2** in kJ mol^{-1} in the high spin state for $Al_2FeO_4^+$ and $Al_2CoO_4^+$, (sextet and quintet) as obtained with different active space sizes. Methodology analogous to ref. 1, compare with Table 2 therein.

	Fe				Co			
	**(5,5)**	**(7,7)**	**(9,9)**	**(11,11)**	**(4,4)**	**(6,6)**	**(8,8)**	**(10,10)**
**CASSCF**	141	142	146	65	284	309	330	314
**CASPT2**	−7	−10	−16	-	60	36	64	8
**NEVPT2**	37	37	40	83	128	86	80	0
**MRCI-D**	66	65	65	56	179	143	146	100
**MRCI-P**	54	53	53	48	163	131	134	87
**MC-PDFT[a]**	54	53	52	72	111	79	0	0

[a] (tPBE)

S5. Additional Computational Results for $AlFe_2O_4^+$ and $AlCo_2O_4^+$

S5.1. Density Functional Theory – Geometric and Electronic Structure

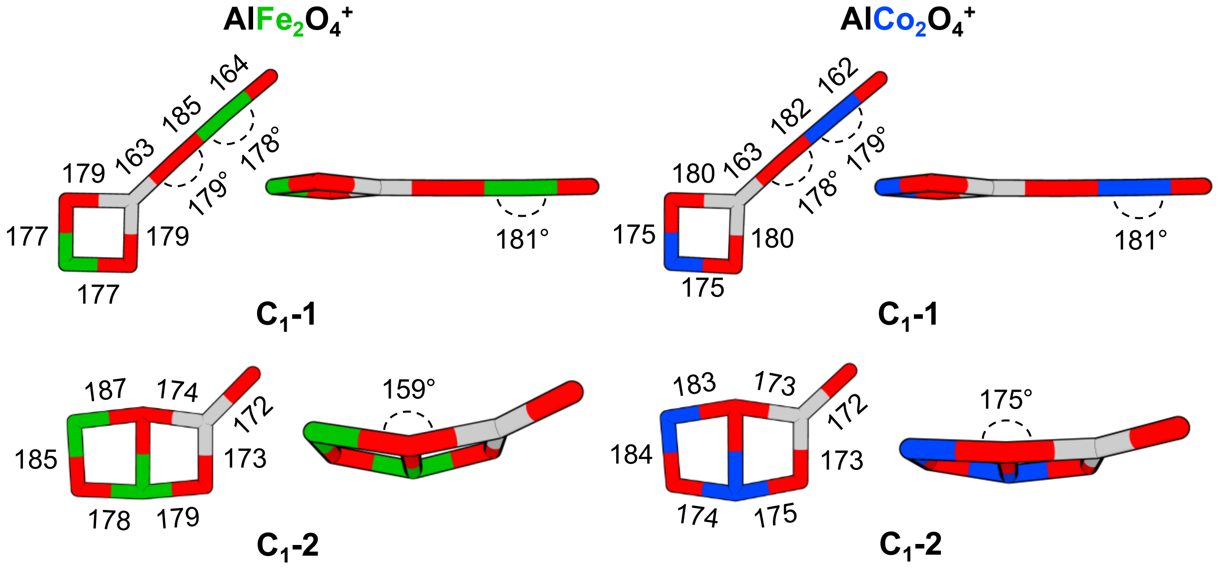

Fig. S6 Bond distances in pm and bond angles of $AlFe_2O_4^+$ and $AlCo_2O_4^+$ **C_1-1** and **C_1-2**. Color code: O - red, Al - gray, Fe - green, and Co - blue.

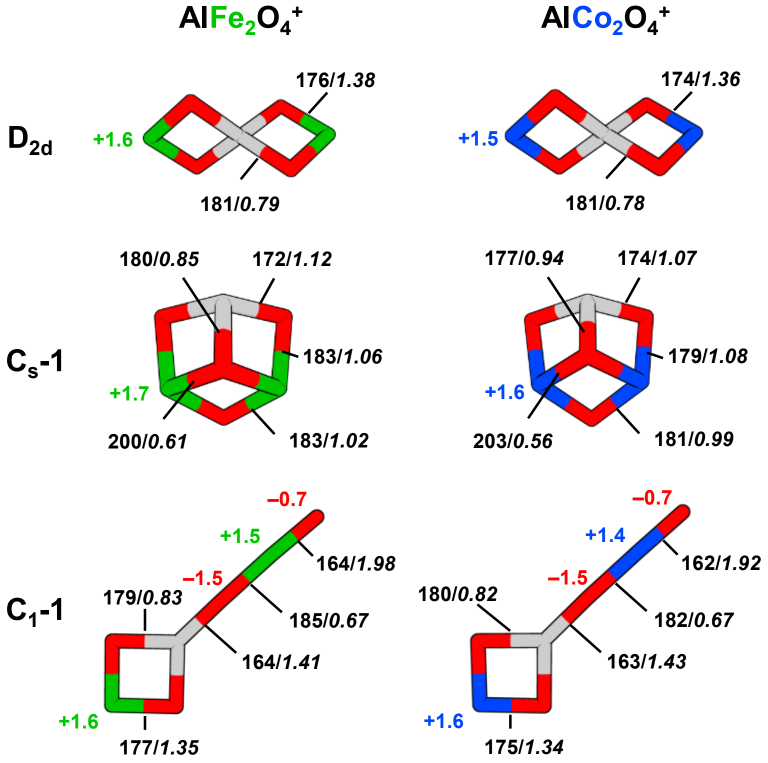

Fig. S7 Bond distances in pm, Wiberg bond analysis (*Italics)* and natural atom population (colored) of $AlFe_2O_4^+$ and $AlCo_2O_4^+$ **D_{2d}**, **C_s-1**, and **C_1-1**. In all structures, the transition metal ions are in a +III oxidation state. Color code: O - red, Al - gray, Fe - green, and Co - blue.

S5.2. Density Functional Theory – Relative Stability

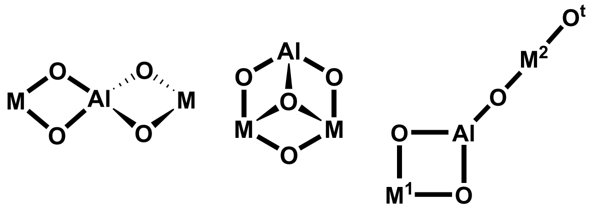

D_{2d} **C_s-1** **C_1-1**

Fig. S8 Structure formulas of $AlFe_2O_4^+$ and $AlCo_2O_4^+$ **D_{2d}**, **C_s-1**, and **C_1-1**. The transition metal ions are symmetry equivalent for **D_{2d}** and **C_s-1**.

Table S4 Relative energies of $AlFe_2O_4^+$ **D_{2d}**, **C_s-1**, and **C_1-1** in kJ mol^{-1} in different spin states (2S+1) as obtained with TPSSh/def2-TZVPP. Values reported with respect to the high spin global minimum (**D_{2d}**) with number of unpaired electrons at transition metal ions (M^1, M^2) according to Fig. S8 in parentheses.

**Isomer**	**2S+1**						
	**11**	**9**	**7**	**7**	**5**	**3**	**1**
**D_{2d}**	0<br>(5,5)	−17<br>(3,5)	70<br>(1,5)	−36<br>(3,3)	52<br>(1,3)	118<br>(1,1)	−1<br>(5,−5)
**C_s-1**	1<br>(5,5)	30<br>(3,5)	63<br>(1,5)	62<br>(3,3)	101<br>(1,3)	132<br>(1,1)	−7<br>(5,−5)
**C_1-1**	96<br>(5,5)	78<br>(3,5)<br>108<br>(5,3)	156<br>(1,5)<br>160<br>(5,1)	89<br>(3,3)	167<br>(1,3)<br>144<br>(3,1)	143<br>(1,1)	96<br>(5,−5)

Table S5 Relative energies of $AlCo_2O_4^+$ **D_{2d}**, **C_s-1**, and **C_1-1** in kJ mol^{-1} in different spin states (2S+1) as obtained with TPSSh/def2-TZVPP. Values reported with respect to the high spin global minimum (**D_{2d}**) with number of unpaired electrons at transition metal ions (M^1, M^2) according to Fig. S8 in parentheses.

**Isomer**	**2S+1**						
	**9**	**7**	**5**	**5**	**3**	**1**	**1**
**D_{2d}**	0<br>(4,4)	2<br>(2,4)	7<br>(0,4)	−65<br>(2,2)	−24<br>(0,2)	17<br>(0,0)	−1<br>(4,−4)
**C_s-1**	14<br>(4,4)	19<br>(2,4)	27<br>(0,4)	38<br>(2,2)	36<br>(0,2)	43<br>(0,0)	−8<br>(4,−4)
**C_1-1**	41<br>(4,4)	4<br>(2,4)<br>101<br>(4,2)	47<br>(0,4)<br>127<br>(4,0)	66<br>(2,2)	109<br>(0,2)<br>93<br>(2,0)	136<br>(0,0)	41<br>(4,−4)

S5.3. Density Functional Theory – Harmonic Vibrational Spectra

Table S6 Calculated wavenumbers ν in cm^{-1} and intensities *I* in km mol^{-1} of the vibrational normal modes of $AlFe_2O_4^+$ and $AlCo_2O_4^+$ **C_1-1** (high spin) as obtained by TPSSh/def2-TZVPP. Nomenclature of transition metal ions according to Fig. S8. Vibrations are categorized in symmetric (s) and antisymmetric (as) or antiphase (ap) stretching (v), and bending (δ) modes.

$AlFe_2O_4^+$			$AlCo_2O_4^+$		
**ν**	***I***	**Assignment**	**ν**	***I***	**Assignment**
1097	623	v_{ap}(Al–O–Fe^2)^a	1067	593	v_{ap}(Al–O–Co^2)^a
893	16	v(Fe^2–O^t)	839	5	v(Co^2–O^t)
760	58	v_s(O–Fe^1–O)^b	714	47	v_s(O–Co^1–O)^b
689	66	v_s(O–Al–O)^c	650	53	v_s(O–Al–O)^c
687	67	v_{as}(O–Fe^1–O)^c	646	36	v_{as}(O–Co^1–O)^c
553	1	v_{as}(O–Al–O)	505	0	v_{as}(O–Al–O)
440	40	δ(Al(–O)$_3$) in-plane	403	38	δ(Al(–O)$_3$) in-plane
328	110	δ(Al(–O)$_3$) out-of-plane	298	104	δ(Al(–O)$_3$) out-of-plane
219	2	δ(Fe^1–O–Al) + δ(Al–O–Fe^2)	209	4	δ(Co^1–O–Al) + δ(Al–O–Co^2)
207	25	δ(Al–O–Fe^2) in-plane	198	26	δ(Al–O–Co^2) in-plane
161	13	δ(Al–O–Fe^2) out-of-plane	164	15	δ(Al–O–Co^2) out-of-plane
75	24	δ(O– Fe^2–O^t) in-plane	108	13	δ(O– Co^2–O^t) in-plane
74	19	δ(O–Fe^2–O^t) out-of-plane	98	8	δ(O–Co^2–O^t) out-of-plane
26	5	δ(O–Fe^2–O^t) out-of-plane	37	0	δ(O–Co^2–O^t) out-of-plane
24	5	δ(O–Fe^2–O^t) in-plane	33	0	δ(O–Co^2–O^t) in-plane

In Table 1: ^{a} c_1/c'_1, ^{b} c_3/c'_3, ^{c} c_4/c'_4

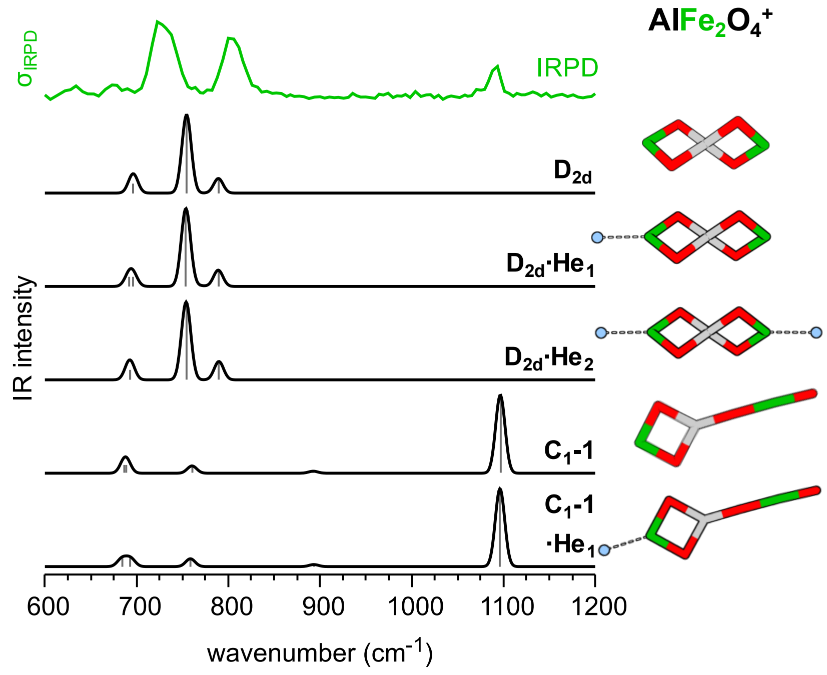

Fig. S9 Experimental IRPD spectrum of $AlFe_2O_4(He_{1,2})^+$ and the corresponding calculated harmonic IR spectra of the bare and He-tagged cations $\mathbf{D_{2d}}$ and $\mathbf{C_1}$**-1** as obtained with TPSSh/def2-TZVPP for the high spin state. Color code: He - light blue, O - red, Al - gray, and Fe - green.

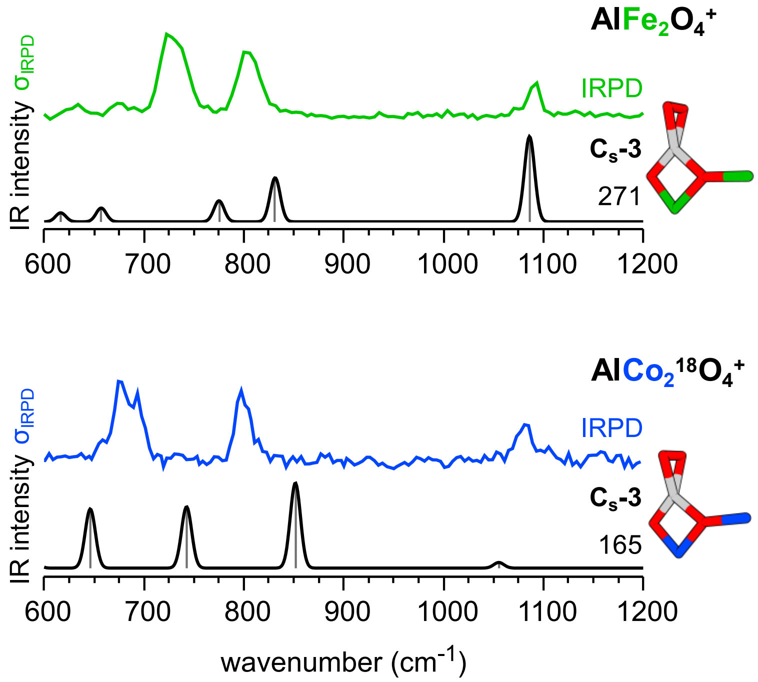

Fig. S10 Experimental IRPD spectra of $AlFe_2O_4(He_{1,2})^+$ and $AlCo_2{}^{18}O_4(He_{1,2})^+$ and corresponding calculated harmonic IR spectra of the bare cation of the most stable superoxide unit containing isomer $\mathbf{C_s}$**-1** as obtained with TPSSh/def2-TZVPP for the high spin state. Relative energies (including zero-point vibrational energies) are given in kJ mol^{-1} with respect to the global minimum structure ($\mathbf{D_{2d}}$). Color code: O - red, Al - gray, Fe - green, and Co - blue.

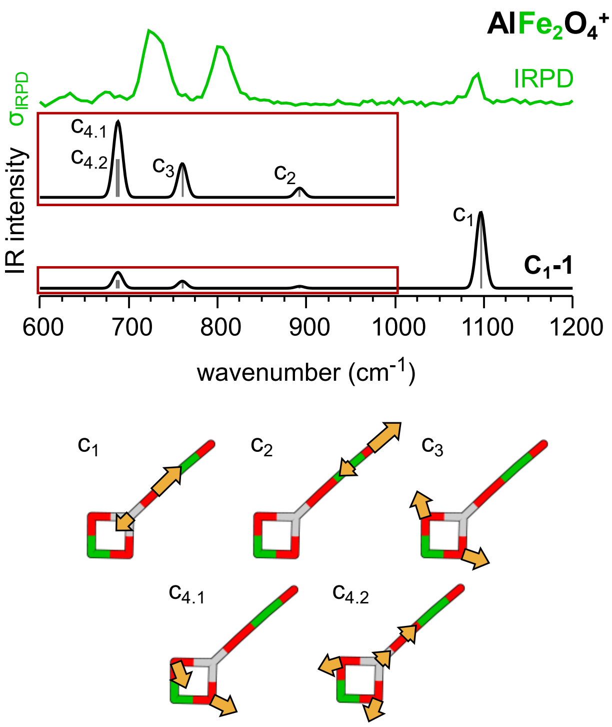

Fig. S11 Experimental IRPD spectra of $AlFe_2O_4(He_{1,2})^+$ as well as calculated harmonic IR spectrum of the bare cation **C_1-1** with an assignment of vibrational normal modes to each peak observed as obtained by TPSSh/def2-TZVPP for the high spin state. Color code: O - red, Al - gray, Fe - green.

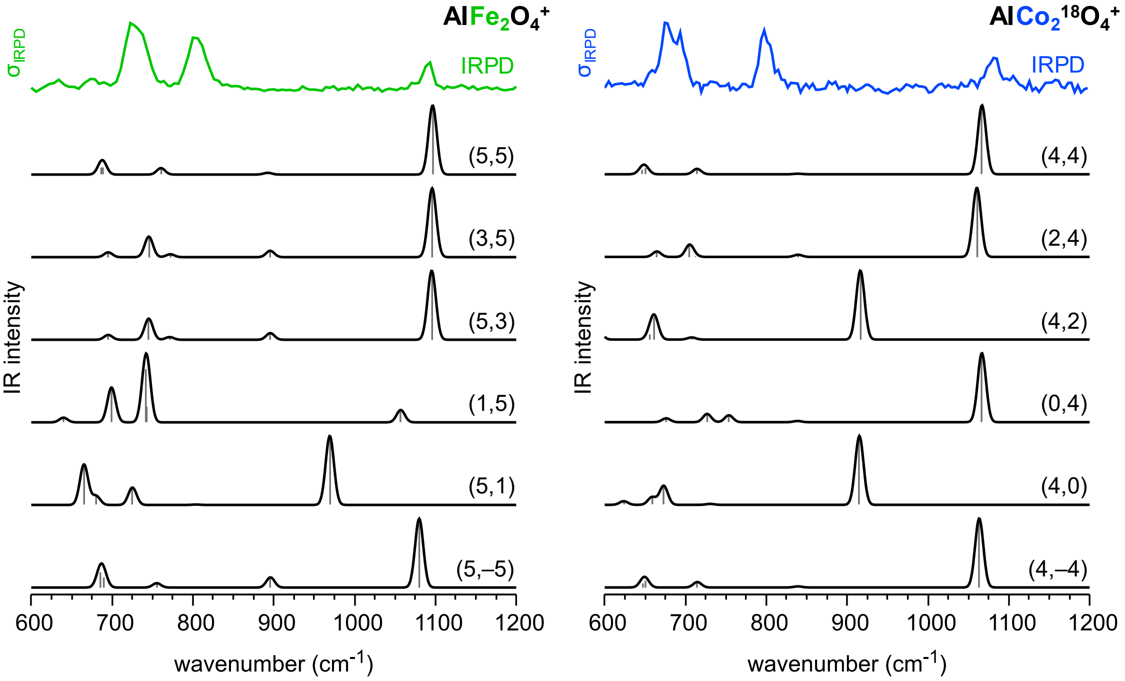

Fig. S12 Experimental IRPD spectra of $AlFe_2O_4(He_{1,2})^+$ and $AlFe_2O_4(He_{1,2})^+$ as well as calculated harmonic IR spectra of the bare cation **C_1-1** in different spin states as obtained with TPSSh/def2-TZVPP. Number of unpaired electrons at transition metal ions (M^1,M^2) according to Fig. S8 in parentheses.

S5.4. Density Functional Theory – Vibrational Spectra by Molecular Dynamics

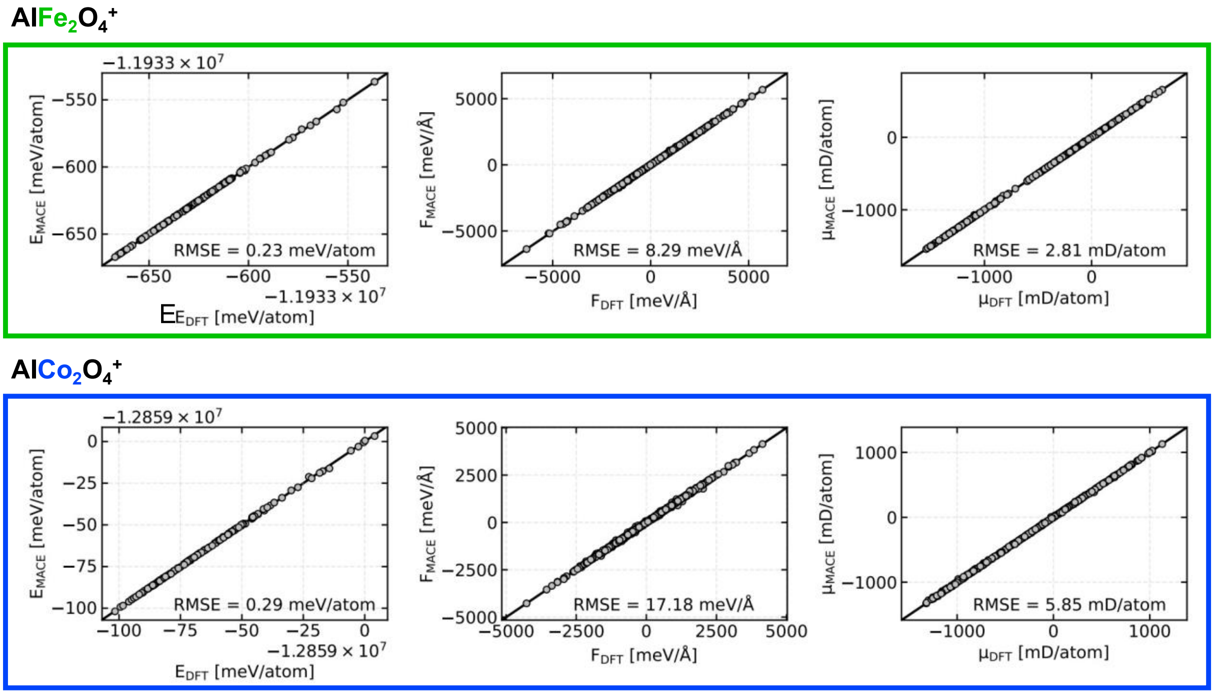

Fig. S13 Parity plots for the energies, forces, and dipole moments obtained by the machine learning interatomic potentials (MLIPs) compared to the reference DFT calculations for **C_1-1** of $AlFe_2O_4^+$ and $AlCo_2O_4^+$.

The MD simulations show a large amplitude motion of the O–M–O^t bending angle which is a consequence of the flat PES for this internal coordinate (see Fig. S17). The peak above 1000 cm^{-1} is associated with an antiphase Al–O–M stretching mode in the nearly linear part of the cluster (see Fig. S11). Structural displacement in internal coordinates along the O–M–O^t bending normal mode reveals that the peak above 1000 cm^{-1} is redshifted for smaller bending angles, however, the corresponding peak intensity is not affected (Fig. S14 and Table S7). This effect counteracts the overestimation of the peak intensity by the harmonic approximation, thereby enhancing the agreement between the predicted and experimental spectra. This effect is more pronounced for Fe compared to Co, since the former exhibits a less steep PES (see Fig. S17).

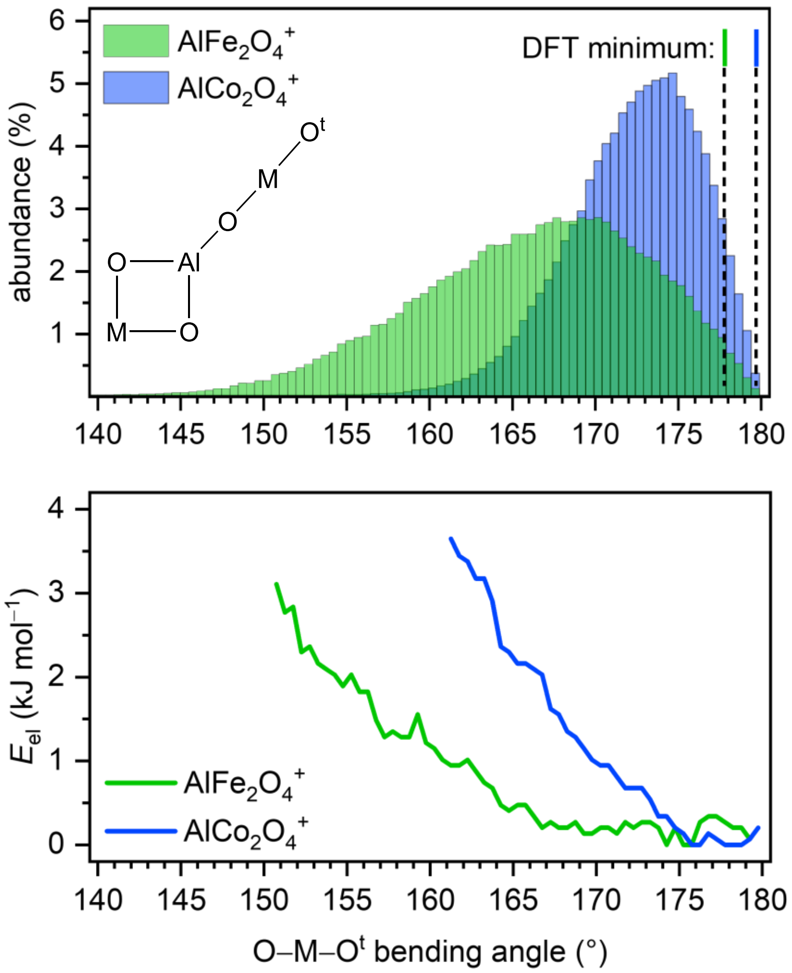

Fig. S14 (top) O–M–O^t bending angle distribution of MD simulation of $AlFe_2O_4^+$ and $AlCo_2O_4^+$ **C_1-1** with (bottom) PBE0$_{MLIP}$ relative energies averaged over 1° of O–M–O^t bending angles.

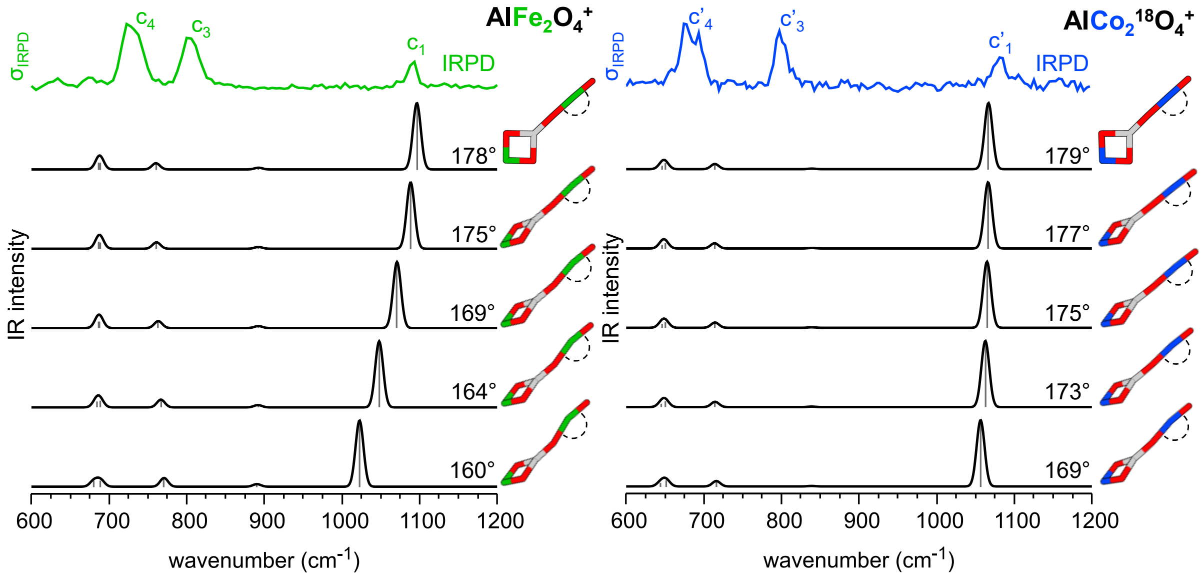

Fig. S15 Experimental IRPD spectra of $AlFe_2O_4(He_{1,2})^+$ and $AlFe_2O_4(He_{1,2})^+$ as well as calculated harmonic IR spectra of the bare cation **C_1-1** with different O–M–O^t bending angles as obtained with TPSSh/def2-TZVPP for the high spin state through structural displacement along bending normal mode in internal coordinates.

Table S7 Calculated wavenumbers ν in cm^{-1} and intensities I in km mol^{-1} of $AlFe_2O_4^+$ and $AlCo_2O_4^+$ **C_1-1** in the high spin state for the vibrations assigned to peak c_1, c_3, and $c_{4.1}$ (two vibrations) in Table 1 as obtained with TPSSh/def2-TZVPP for different O–M–O^t bending angles.

O–M–O^t angle (°)	c_1		c_3		$c_{4.1}$		$c_{4.2}$	
	ν	I	ν	I	ν	I	ν	I
**$AlFe_2O_4^+$**								
178	1096	623	760	58	688	66	686	67
175	1088	623	761	60	688	64	686	67
169	1071	622	763	65	687	62	686	65
164	1048	619	766	72	688	62	683	60
160	1022	610	770	80	689	62	680	55
**$AlCo_2O_4^+$**								
179	1066	593	714	46	650	53	646	36
177	1066	592	714	46	650	53	646	36
175	1064	586	714	46	650	53	645	35
173	1062	577	714	46	650	53	645	35
169	1056	555	716	45	651	54	644	35

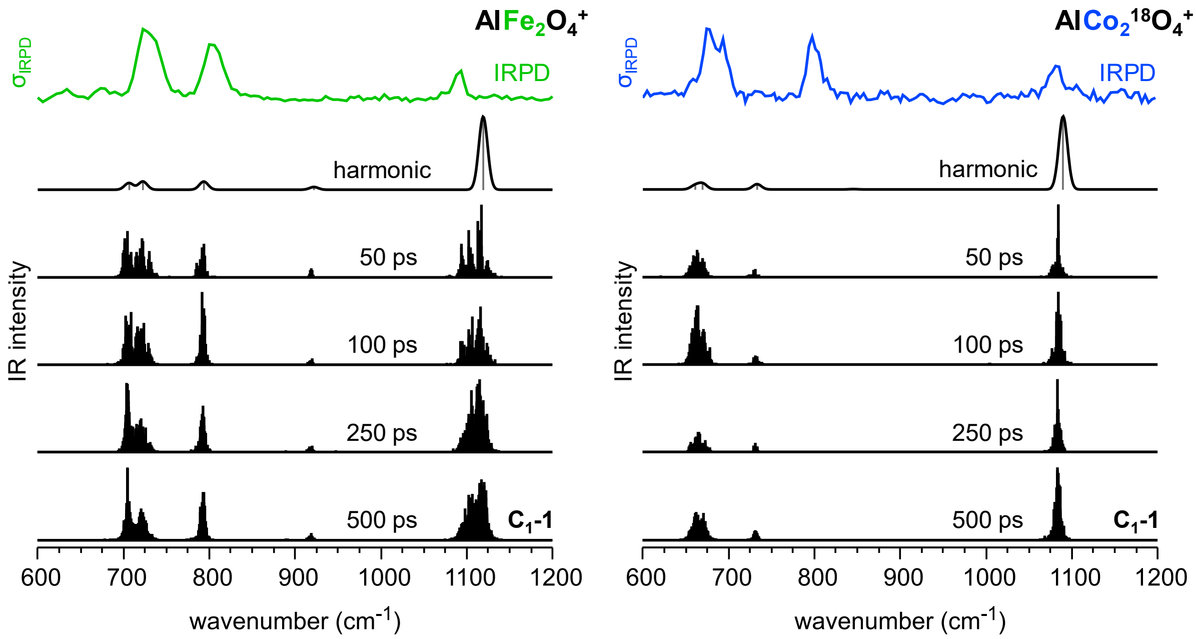

Fig. S16 Experimental IRPD spectra of $AlFe_2O_4(He_{1,2})^+$ and $AlCo_2{}^{18}O_4(He_{1,2})^+$ compared to IR spectra of the bare cation **C_1-1** obtained with different simulation methods: harmonic approximation (PBE0/def2-TZVPP) as well as MD simulations with different simulation times (PBE0-quality MLIP). Color code: O - red, Al - gray, Fe - green, and Co - blue.

Table S8. Ratio of integrated peak areas of the IRPD spectrum of $AlFe_2O_4^+$ and $AlCo_2O_4^+$ as well as the harmonic and MD-based IR spectra of **C_1-1**.

	$AlFe_2O_4^+$			$AlCo_2O_4^+$		
	**IRPD**	**harmonic**	**MD**	**IRPD**	**harmonic**	**MD**
**c1/c3**	0.3	10.8	4.4	0.6	12.7	8.8
**c1/c4**	0.2	4.7	1.9	0.3	6.6	1.2

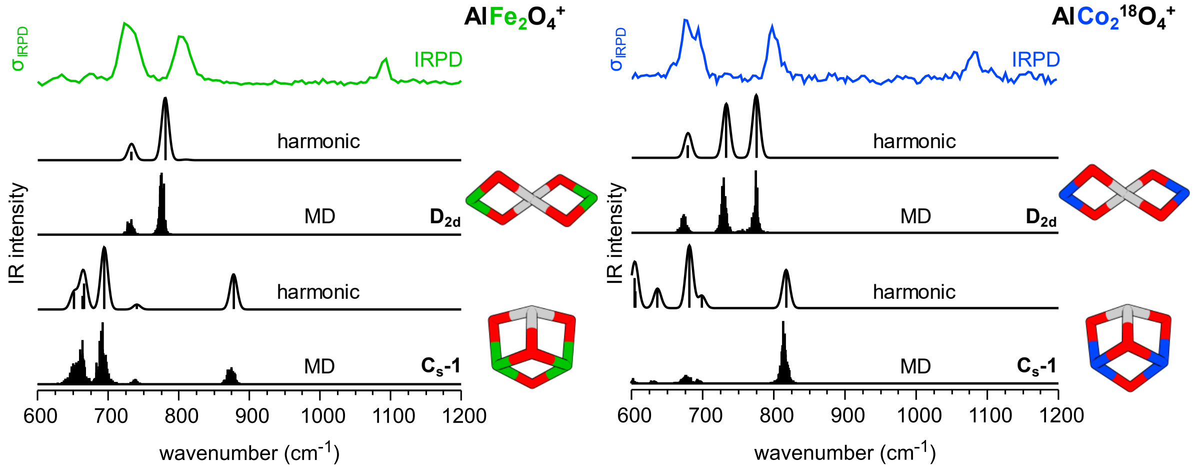

Fig. S17 Experimental IRPD spectra of $AlFe_2O_4(He_{1,2})^+$ and $AlCo_2{}^{18}O_4(He_{1,2})^+$ as well as harmonic IR spectra as obtained with PBE0/def2-TZVPP together with $PBE0_{MLIP}$ quality MD-based IR spectra of the bare cations **D_{2d}**, **C_s-1**. Color code: O - red, Al - gray, Fe - green, and Co - blue.

S5.5. Density Functional Theory – Reactivity Calculations

All adsorption and reaction energies (including zero-point vibrational energies) are calculated for the high spin state. Three potential adsorption sites for **C_1-1** are identified: the Al atom (−34 kJ mol^{-1}), the nearly linear coordinated transition metal atom (Fe: −16 kJ mol^{-1}; Co: −18 kJ mol^{-1}), and the rectangularly coordinated transition metal atom (Fe: −75 kJ mol^{-1}; Co: −86 kJ mol^{-1}). Given that the adsorption enthalpy for the rectangularly coordinated transition metal atom is the most exothermic, this encounter complex serves as the reactant for our investigation of the C–H bond activation. As the transition metal ions are already in an oxidation state of +III, oxidative addition is unlikely to occur. In a previous study, Li *et al.* demonstrated a C–H bond activation of methane and formaldehyde formation via an adjacent Lewis acid-base pair for $AuV_2O_6^+$, containing an Au(+III)-atom.[2] Given the similar oxidation states between $AuV_2O_6^+$ as well as $AlFe_2O_4^+$ and $AlCo_2O_4^+$, we considered an analogous reaction pathway. However, none of the employed functionals predicts a negative apparent barrier for this reaction mechanism (see Fig. S18)

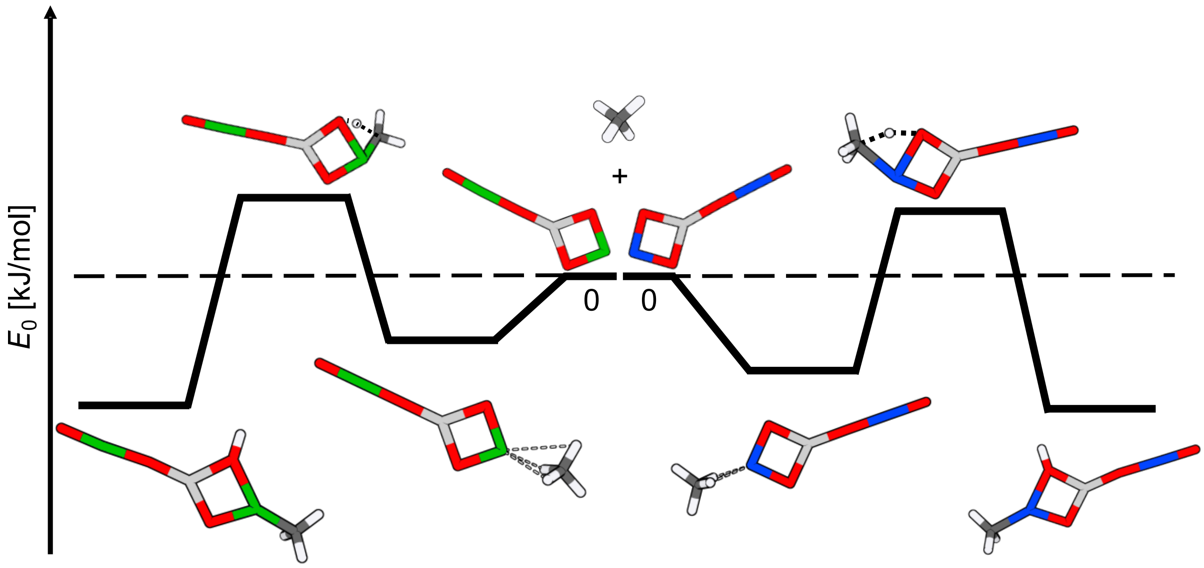

–114	22	–81	**PBE**	–94	4	–130
–116	44	–75	**TPSSh**	–86	29	–141
–141	24	–85	**PBE0**	–93	16	–169
–170	48	–81	**BHLYP**	–70	36	–187

Fig. S18 Calculated C–H bond activation pathway for the interaction of methane with $AlFe_2O_4^+$ and $AlCo_2O_4^+$ **C_1-1** as obtained with TPSSh/def2-TZVPP for the high spine state. Color code: H - white, C - gray, O - red, Al - gray, Fe - green, and Co - blue.

References

[1] Müller, F.; Stückrath, J. B.; Bischoff, F. A.; Gagliardi, L.; Sauer, J.; Debnath, S.; Jorewitz, M.; Asmis, K. R., Valence and Structure Isomerism of Al2FeO4+: Synergy of Spectroscopy and Quantum Chemistry. *J. Am. Chem. Soc.* **2020,** *142*, 18050-18059, https://doi.org/10.1021/jacs.0c07158.

[2] Li, Z.-Y.; Li, H.-F.; Zhao, Y.-X.; He, S.-G., Gold(III) Mediated Activation and Transformation of Methane on Au1-Doped Vanadium Oxide Cluster Cations AuV2O6+. *J. Am. Chem. Soc.* **2016,** *138*, 9437-9443, https:// doi.org/10.1021/jacs.6b03940.
